# 3D Hyperbolic Kirigami Metamaterials With Tunable Auxeticity and Multistability

**DOI:** 10.1002/advs.202506703

**Published:** 2025-06-23

**Authors:** Yu Lei, Yan Wang, Ruizhi Cui, Xiaolong Huang, Lei Zhang, Yuan Jin, Jinling Gao, Biwei Deng

**Affiliations:** ^1^ Yongjiang Laboratory Ningbo Zhejiang 315202 China; ^2^ School of Mechanical Engineering and Mechanics Ningbo University Ningbo Zhejiang 315211 China; ^3^ State Key Laboratory of Mechanics and Control for Aerospace Structures Nanjing University of Aeronautics and Astronautics Nanjing 210016 China; ^4^ MIIT Key Laboratory of Multifunctional Lightweight Materials and Structures Nanjing University of Aeronautics and Astronautics Nanjing 210016 China

**Keywords:** auxeticity, hyperbolic tessellations, kirigami metamaterials, multistability

## Abstract

Kirigami mechanical metamaterials provide exceptional tunability in mechanical properties and morphing capabilities, exhibiting great potential for deployable and actuatable devices. However, most kirigami structures can only deform freely within a 2D plane, with limited out‐of‐plane deformability, making them inadequate for constructing periodic objects with arbitrary 3D shapes. Here, a novel class of 3D mechanical metamaterials with hyperbolic kirigami tessellations has been developed. By projecting hyperbolic kirigami templates onto three types of triply periodic minimal surfaces, candidate structures are developed with remarkable properties. An extreme negative Poisson's ratio of ‐1 and tunable mechanical multistability are uncovered through theoretical analysis, numerical simulations, and experiments thanks to the flexible kirigami geometry. Notably, the structure achieves a maximum volume expansion of up to 488% during auxetic morphing. Furthermore, programmable morphing behaviors are demonstrated through voxelated assemblies of kirigami unit cells with varying geometrical parameters. The novel design strategy presented in this work based on hyperbolic kirigami tessellations opens up new avenues toward auxetic and multistable mechanical metamaterials with broad applications spanning shape‐morphing architectures, deployable space structures, and soft machines.

## Graphical abstract

This study proposes a hyperbolic tessellation‐based structure that integrates TPMSs (Triply Periodic Minimal Surfaces, including SP: Schwarz Primitive, SD: Schwarz Diamond, and GY: Gyroid) with rotating rigid polygons, which enables 3D scalable deformation. The structure exhibits auxeticity and multistability, with a maximum volume expansion rate of up to 488%. This design provides a novel approach for developing reusable, deployable, and programmable 3D metamaterials.

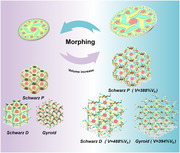

## 1 Introduction

Kirigami, traditionally known as the art of paper cutting, has been rediscovered in the research frontiers of mechanical metamaterials in the past decade.^[^ ^1^, ^2^, ^3^, ^4^, ^5^ ^]^ Numerous unique devices with exceptional mechanical responses have been realized through kirigami designs, such as deployable transformers,^[^ ^6^, ^7^, ^8^, ^9^ ^]^ robotic actuators,^[^ ^10^, ^11^, ^12^ ^]^ flexible electronics,^[^ ^13^, ^14^, ^15^, ^16^ ^]^ optomechanical devices,^[^ ^17^, ^18^, ^19^, ^20^ ^]^ etc. A key merit of kirigami structures is the capability of morphing into 3D shapes from a 2D plane under specific mechanical stimuli.^[^ ^21^, ^22^, ^23^ ^]^ Such morphing capability is programmed by modulating the out‐of‐plane bending or torsion of 2D elements in kirigami.^[^ ^17^, ^18^, ^19^ ^]^ However, when 3D periodic objects with arbitrary volumes need to be produced, the morphing capability of kirigami metamaterials falls inadequate. The dominant deformation behavior of kirigami structures is the coordinated rotation of periodic elements in the in‐plane dimensions. Thus, they can achieve large expansions or reductions in size within a 2D plane, while the morphable range into the out‐of‐plane dimension is very limited.^[^ ^24^ ^]^ Therefore, most reported kirigami structures are quasi‐3D. Recent reports on discrete kirigami units have demonstrated their deployment in finite 3D volumes,^[^ ^7^, ^25^ ^]^ however, these finite structures are bound by incompatible boundaries or twist behavior beyond Cauchy elasticity, which restrain them from periodically extension in all three dimensions. The lacking of 3D extendable kirigami designs greatly hindered the broader applications of kirigami metamaterials, such as energy absorption, deployable pop‐up structures, and soon, where large volumetric morphing is in demand.

In a typical kirigami design, a 2D plane is tessellated with periodic elements, while adjacent elements are hinged at their corners to moderate rotational deformations.^[^ ^26^, ^27^, ^28^ ^]^ The rotation‐driven coordinated morphing of kirigami structures dictates their unique mechanical properties, such as auxeticity^[^ ^29^, ^30^, ^31^ ^]^ and multistability.^[^ ^25^, ^27^, ^32^, ^33^ ^]^ The essence of research on kirigami‐based metamaterials lies within the devising strategy of the kirigami geometry. Modern computational tools such as machine learning^[^ ^34^ ^]^ have been used to realize optimized mechanical responses. Many theoretical works have been developed on how to design these 2D tessellations to realize 3D morphing.^[^ ^35^, ^36^, ^37^, ^38^, ^39^ ^]^ For instance, researchers have discovered that a 2D tessellation can be inversely designed for the kirigami once arbitrary final shapes, no matter in 2D or 3D, are designated.^[^ ^40^, ^41^ ^]^ However, these theories of constructing kirigami generally assume the initial 2D state to be Euclidean. Such assumption is dominant mostly due to the considerations on manufacturability. Theoretical works have shown that a non‐Euclidean 2D plane can accommodate kirigami tessellations as well.^[^ ^42^ ^]^ In fact, non‐Euclidean planes can be constructed in the Euclidean 3D space simply with non‐zero curvatures, and then actually be manufactured, thanks to additive manufacturing.^[^ ^43^, ^44^, ^45^, ^46^ ^]^ In another word, it is possible to make kirigami structures that morph from one 3D state to another with significant volumetric change. Compared with 3D morphable structures with hinged solid polyhedra,^[^ ^33^ ^]^ kirigami metamaterials based on non‐Euclidean 2D planes are potentially lighter in weight and offer the flexibility in manufacturing. Nevertheless, a suitable non‐Euclidean 2D system as the design guideline is needed.

In light of this problem, an intriguing non‐Euclidean 2D system is the hyperbolic ones that take shape as triply periodic minimal surfaces (TPMSs) in a Euclidean 3D space.^[^ ^47^ ^]^ They have unique hexagonal symmetries that allow a variety of different tessellations. So far, TPMSs have been a rather effective platform for the creations of new mechanical metamaterials.^[^ ^48^, ^49^, ^50^, ^51^ ^]^ Their flexible design, comprehensive mechanics, and convenient manufacturability are widely recognized. Recently, generations of metamaterials based on the concept of geometrical tessellations have emerged. For example, by segregating phases according to TPMS tessellations, one can design mechanical metamaterials with tunable elastic properties.^[^ ^52^ ^]^ While mechanical metamaterials based on TPMSs have been investigated for their stiffness,^[^ ^53^, ^54^ ^]^ strength,^[^ ^55^, ^56^ ^]^ energy absorption,^[^ ^57^, ^58^ ^]^ etc., their potential in reconfiguration of shapes remains elusive. Till now, tessellating TPMSs into morphable kirigami metamaterials has not yet been explored.

In this work, we demonstrate the full process starting from designing hyperbolic kirigami tessellation to building 3D mechanical materials with programmable morphing capability. Under our design strategy, a conjugate family of 3D mechanical materials is realized with tunable stress‐strain responses. With coordinated 3D rotations of the hinged elements in the deformed mechanical metamaterials, unconventional mechanical properties such as auxeticity and multistability are exhibited. The Poisson's ratio of the realized mechanical metamaterials are negative and can reach down to ‐1. Furthermore, the synthetic generation of voxelated metamaterial devices are explored. Differently, parameterized unit cells are compounded to form metamaterial blocks of desired mechanical responses. As a proof‐of‐concept study, we demonstrated that non‐Euclidean hyperbolic space is an ideal bridge connecting 2D concept with 3D reality in the construction of new metamaterials. Overall, our work is pioneering the unvisited designs of hyperbolic kirigami as a novel platform for discovering 3D kirigami metamaterials with unique mechanical properties.

## 2 Results and Discussion

### 2.1 Design of 3D Hyperbolic Kirigami Metamaterials

**Figure 1:**
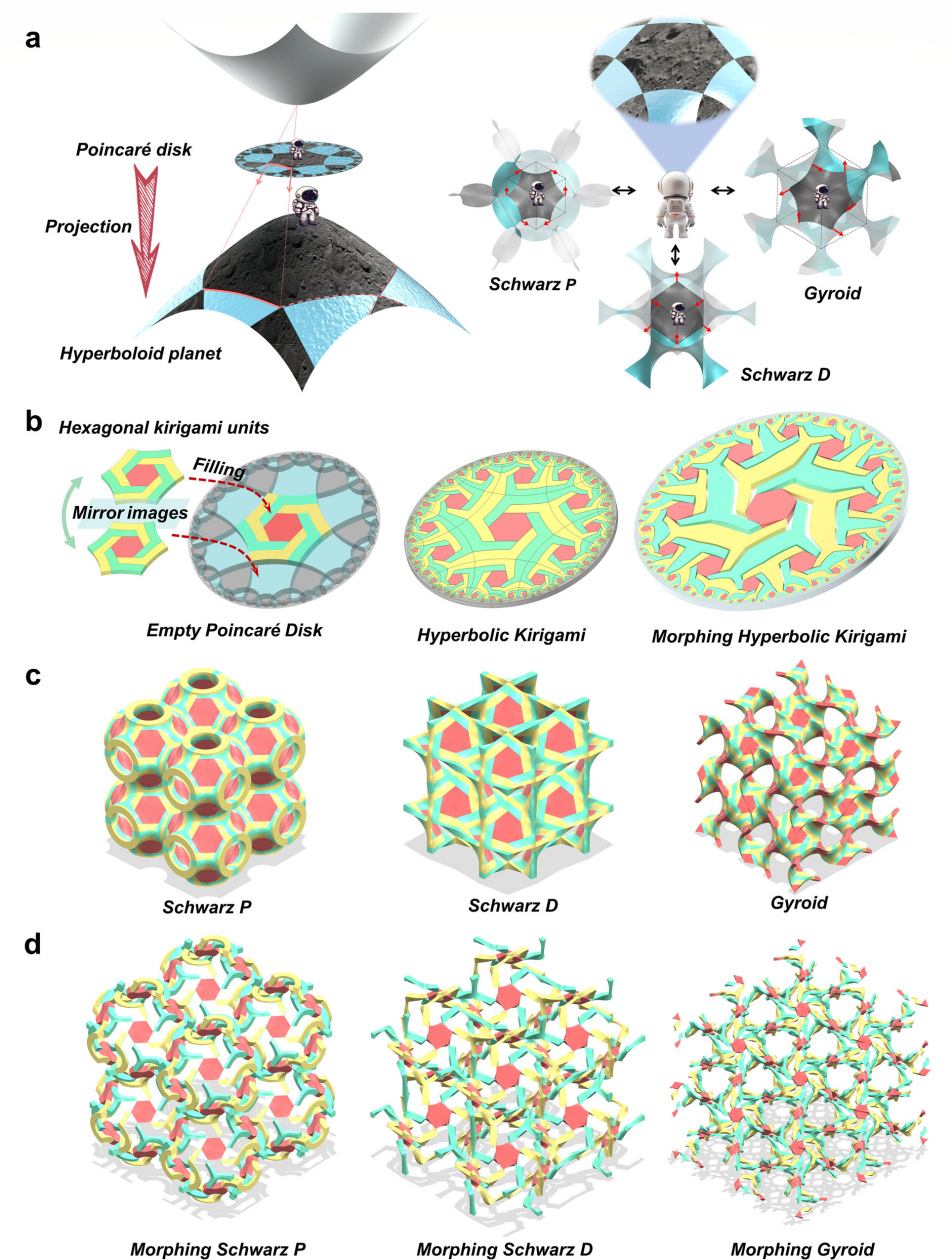
Hyperbolic tilings projected onto TPMS. a) The hyperbolic 2D plane is represented in a Poincaré disk using a hexagonally‐symmetric design. This tiling is projected onto the unit cells of the SP, SD, and GY surfaces. These configurations can be interchanged via a Bonnet rotation, with red arrows indicating the normal direction, and all share the same hexagonal tiling property. b) The disk is tessellated with identical, non‐Euclidean hexagons that rotate and expand while retaining their shape. Each hexagon is hinged to six boomerang‐shaped arms and mirrors adjacent units. Every hexagonal unit is a mirror image of its neighbor. c) and d) The hyperbolic kirigami tessellations are realized through three types of multi‐cell TPMSs, each exhibiting auxetic properties.

The tessellations of a hyperbolic 2D plane can be explicitly depicted in a Poincaré disk. As shown in **Figure** **1**a, when projecting a hyperboloid surface toward a single vertex on the center axis, all points from the hyperboloid surface can be identically mapped on the Poincaré disk midway. The same goes for other 3D surfaces, such as TPMSs, which are compatible with similar projections on the Poincaré disk. In particular, a hexagonal tessellation of the Poincaré disk can be projected onto three conjugate TPMSs: Swartz Primary (SP), Swartz Diamond (SD) and Gyroid (GY). Any of these surfaces can be converted into another via a Bonnet rotation.^[^ ^59^ ^]^ Since all points on the conjugate TPMSs are identically mapped on the Poincaré disk, from a perspective on the hyperbolic 2D space, it is possible to distinguish the three differently projected hexagonal tessellations. For instance, each vertex is shared by four hexagonal surface units, which is proven to be a key symmetrical merit in realizing compatible morphing later on, regregardless of whether the tessellation is performed on SP, SD or GY. To construct our kirigami metamaterials, the hexagon units of the unique Poincaré disk are further divided. These hexagonal kirigami units by design are able to rotate and expand while maintaining their hexagon shapes as shown in Figure 1b. The underlying mathematical description of their hyperbolic tessellation is presented in Figure S1 (Supporting Information). In each unit, the inner hexagon is hinged at its corners to six boomerang‐shaped arms. Every hexagonal kirigami unit filled in the empty Poincaré disk is the mirror image of its neighboring unit. Therefore, adjacent units have opposite rotating directions. By merging together 4 rotatable arms from 4 adjacent kirigami units, a harmonious morphing is guaranteed since all units are able to rotate simultaneously. In Figure 1c, the designed hyperbolic kirigami tessellations are embodied by three types of TPMSs, which periodically occupy Euclidean 3D space. These tessellated TPMSs, as 3D kirigami metamaterials, are expected to morph in a resembling manner compared to the original hyperbolic kirigami, as depicted in Figure 1d. The mere difference is that the hyperbolic kirigami only allows in‐plane rotations, due to its 2D nature, while the tessellated TPMSs accommodate out‐of‐plane deformations as well.

### 2.2 Auxeticity and Multistability of 3D Kirigami Metamaterials

**Figure 2:**
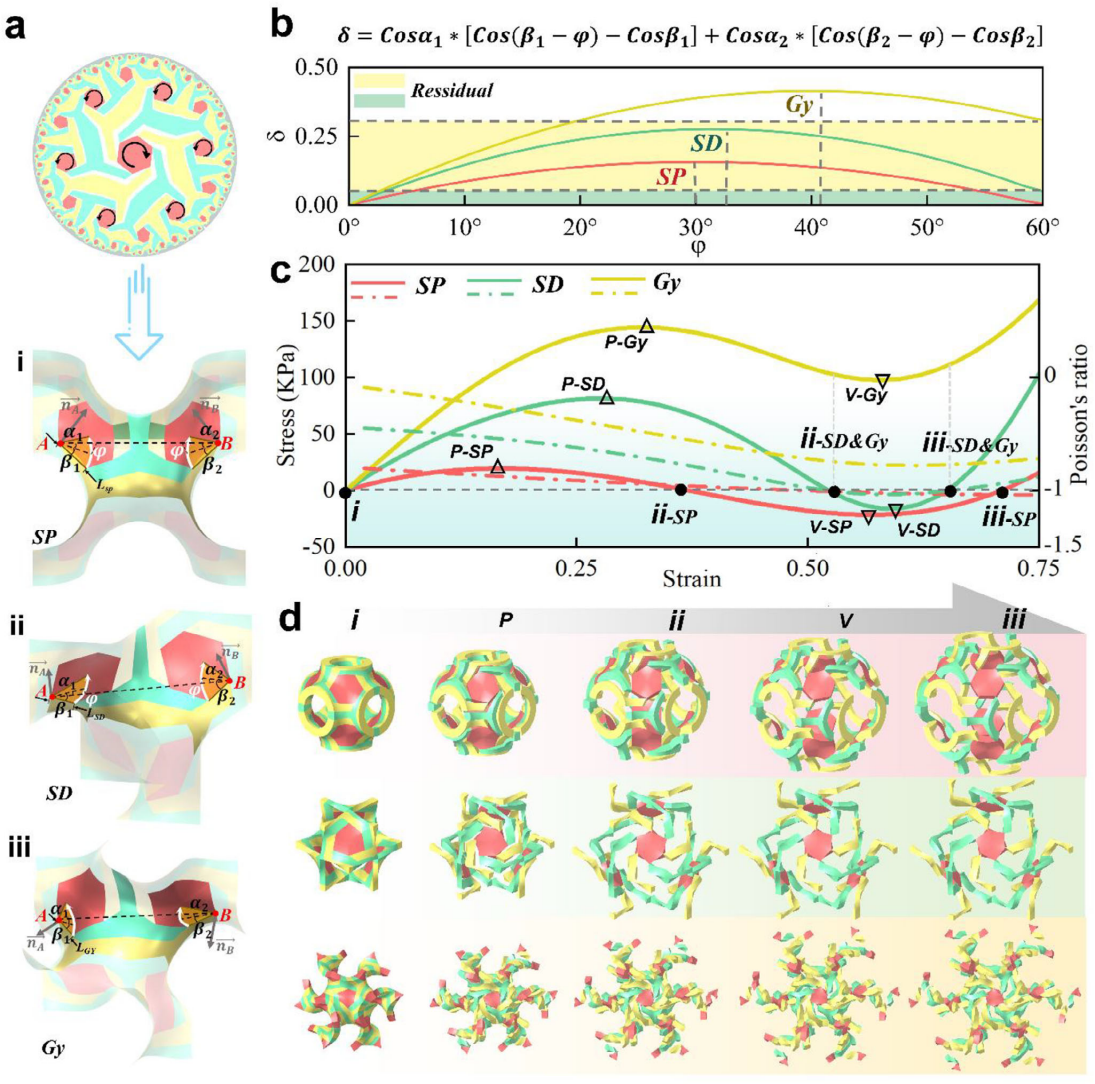
Mechanical response of multistable auxetics to uniaxial loading. a) The deformation of a hexagonal rigid rotational configuration in hyperbolic space and its geometric reconstruction in 3D Euclidean space through three types of TPMS. b) The relationship between the dimensional constraint factor (*δ*) and the rotation angle (𝜑).In the 3D configuration, the rotating unit is a hexagonal shape, the range of 𝜑 to 0°–60°.c) FEA (solid lines) stress‐strain curves during loading (tension) and characterized Poisson's ratio (dashed lines) for SP (red), SD (green) and GY (yellow), respectively. d) The inset figures represent the auxetic states at different stages (i, p, ii, v, and iii). Here, i represents the first stable state. For the SP and SD structures, iii corresponds to the second stable state, while ii marks the critical tipping point from the first stable state to the second. In contrast, the GY structure exhibits only a single stable state.

Like many planar kirigami metamaterials which morph by internal rotations,^[^ ^39^, ^60^ ^]^ our 3D kirigami metamaterials are designed to be auxetic and potentially multistable. Moreover, the new design of kirigami metamaterials leads to more deformation mechanisms to be discovered. Their dimension expansions are closely monitored within the selected representative volume elements (RVEs), which are the cubic unit cells of each 3D kirigami. **Figure** **2**a illustrates the harmonious rotation of a hyperbolic kirigami and its projections on three types of TPMSs. Inspired by the known bistable 2D kirigami (Figure S2, Supporting Information), the key factor determining the bistability of our 3D kirigami structures is the evolution of length AB (L_AB_) as the harmonious rotation occurs. During the deformation process, with the yellow arm fixed, the red rotating body rotates 60° around points A and B, driven by the green arm. The rotation axis is defined by the normal vectors (denoted as ${\vec{n}}_{A}$ and ${\vec{n}}_{B}$) at points A and B on the surface. As the rotating body moves, each edge sweeps out a sector‐shaped area. Since points A and B are fixed on the yellow arm, the rotation of the body is constrained. The degree of this constraint is quantified by the change in the distance between A and B, which can be expressed as: *L_AB_* = L*δ, where L is the edge length of the rotating body, and δ is the dimensional constraint factor. Due to differences in geometric configurations, the edge lengths of the red rotating bodies on the three surfaces vary, necessitating the use of δ as a unified evaluation criterion. Figure 2b illustrates the dimensional constraint factor is given by: δ  =  *Cos*α_1_*[*Cos*(β_1_ − φ) − *Cos*β_1_] + *Cos*α_2_*[*Cos*(β_2_ − φ) − *Cos*β_2_], where 𝛼₁ and 𝛼_2_ are the projection angles of the line AB onto the sector‐shaped rotation area, and 𝛽₁ and 𝛽_2_ are the angles between the projected line of AB and the initial rotating edge. The sizes of these angles are fixed in the three configurations, with specific values listed in Table S1 (Supporting Information). 𝜑 is the rotational angle. The increasing interval of δ represents the energy storage process, where external work is converted into strain energy, and the external load force is positive. Conversely, the decreasing interval of δ corresponds to the autonomous release of strain energy, during which the external load force is negative. Figure 2c presents the evolution of effective stress and Poisson's ratio when the unit cells from the three kirigami metamaterials are subject to uniaxial tension along the orthogonal direction, or the [100] direction in their cubic symmetrical system. Since the Poisson's ratio of an anisotropic material could vary depending on the orientation of deformation, to avoid any ambiguity, the Poisson's ratios discussed in this work are all based on the [100] direction if not specified otherwise.

Here, 3D kirigami metamaterials demonstrate two distinct types of stress‐strain responses: bistable snap‐through with a negative local stress minimum (SP and SD) and monostable snap‐through with a positive local stress minimum (GY) in the stress–strain relationship. Notably, the monostable state cannot retain its deformed shape after the load is removed, whereas the bistable state, characterized by two stable configurations, is capable of maintaining its transformed shape even after the deforming force is released. By definition, multistability implies the presence of multiple distinct stable states within the designed structure. Due to the mirror symmetry of the SP structure (Figure S3, Supporting Information), *δ* increases from 0, reaches a maximum, and then returns to 0, exhibiting symmetry ≈𝜑 = 30°. This leads to the symmetric behavior observed in the stress‐strain curve of SP. The point iii‐SP represents the second stable state of SP, which, like the first stable state (i‐SP), exhibits a stress‐free condition as shown in Figure 2d and Figure S4 (Supporting Information). In contrast, SD and GY structures exhibit poorer mirror symmetry, leading to the generation of additional strain energy during the morphing process beyond that caused by *δ*. Furthermore, the *δ* values of SD and GY do not return to 0 when 𝜑 reaches 60°. Specifically, GY exhibits a relatively higher residual *δ*, which results in the disappearance of the bistability phenomenon. On the other hand, SD retains a residual *δ*, leading to an internal stress distribution even in its second stable state. Thus, the true (effective) stress distributions in the critical regions of SP, SD, and GY structures – with particular focus on the hinges where stress concentration occurs – are non‐uniform (Figure S4; Figure S5, Supporting Information). Furthermore, we discovered that the Poisson's ratio stays above ‐1 when the stress is positive, and falls below ‐1 as soon as the stress falls below zero (SP and SD). Although the ideal morphing of the 3D kirigami grants equal expansion in all three axes, meaning a Poisson's ratio of ‐1, the uniaxial loading causes anisotropic distribution of bent arm elements due to the paths of force transmission. Similar observation has been made in reported 2D kirigami cases as well.^[^ ^27^, ^61^ ^]^ On the other hand, for the GY case, where the stress remains positive during stretching in most instances, the Poisson's ratio stays greater than ‐1. The morphing process involves two coupled mechanical responses: i) global rigid‐body rotation, which governs the auxetic effect, and ii) localized small‐strain deformations at hinges, which contribute to elastic energy accumulation and give rise to multistability. These two behaviors are intrinsically linked through the dimensional constraint factor δ. Thus, the simultaneous realization of strong auxeticity and mechanical multistability in the hyperbolic kirigami design is fundamentally enabled by this rotational mechanism.

### 2.3 Effect of Design Parameters on Mechanical Properties

**Figure 3:**
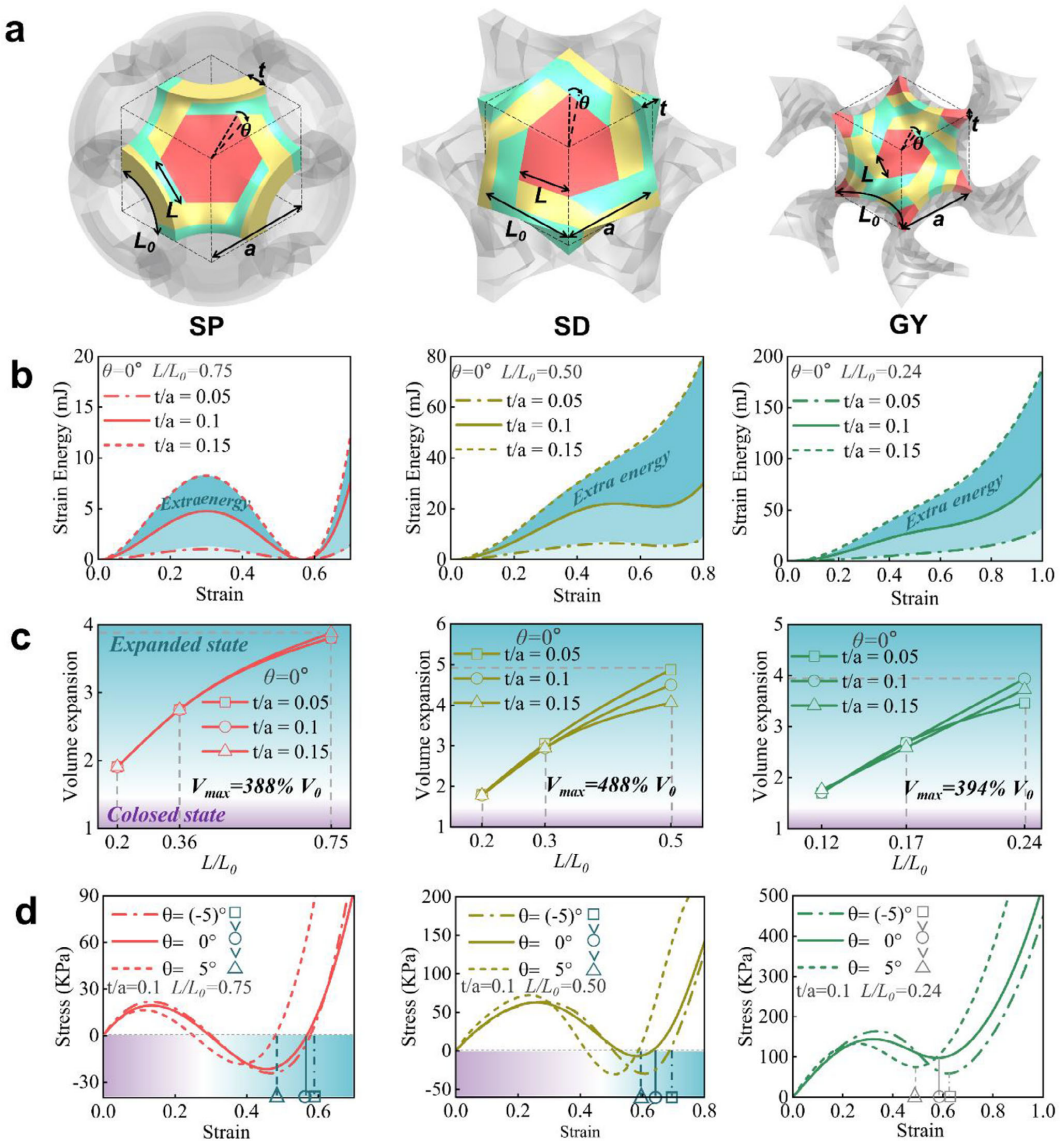
The hexagonal rotating rigid unit configuration results from the evolution of TPMSs (SP, SD, and GY), where the red hexagon represents the rotating part, and the yellow and green parts are connecting arms, all linked to the red hexagon through hinges a). The effect of the design parameter (*t/a*) on strain energyb); the influence of the design parameter (*L/L_0_*) on the volumetric expansion ratio (c) and the impact of the design parameter (*θ*) on stress‐strain of the three configurations (SP, SD and GY) (d).

As an important feature of our kirigami design, the morphing behavior and mechanical properties can be flexibly tuned by adjusting the geometry of the starting hexagon kirigami unit. As shown in **Figures** **3**a and S6 (Supporting Information), the thickness of the hyperbolic plates (*t/a*), the ratio of the inner hexagon size over the width of the boomerang‐shaped arm (*L/L_0_*), and the initial angle between the inner hexagon and the hexagonal kirigami unit (*θ*) are varied to demonstrate tunable geometrical designs of the SP, SD, and GY kirigami metamaterials. It is shown that all SP kirigami metamaterials exhibited a bistable mechanical behavior. This phenomenon is also primarily attributed to the inherent mirror symmetry of the SP structure, The effect of the design parameter (*t/a*) on strain energy is studied, as shown in Figure 3b. An increase in the *t/a* ratio leads to amplified local maximum and minimum stresses during bistable morphing. This is because the elastic energy stored in the rotating arms increases with greater thickness. In addition, for the two structural components adjacent to the hinge, besides axial rotation, there is also a tendency for mutual twisting and bending due to the increased wall thickness as illustrated in Figure S7 (Supporting Information). As a result, the strain energy gradually increases as the thickness increases in SP, SD, and GY kirigami metamaterials. Figure 3c illustrates the influence of the inner hexagon size ratio on the bistability behavior of the rotating configuration. An increase in the *L/L_0_* ratio allows the metamaterial to stretch over a larger strain during its bistable morphing. This is because the size of the inner hexagon plate determines the displacement its rotation brings to the morphed kirigami unit. With the design parameters fixed at *t/a* = 0.05, 0.1, 0.15, and *θ* = 0°, the measured volumetric expansion rate of the SP, SD, and GY structures gradually increases as *L/L_0_* increases. The study revealed that when the design parameter *L/L_0_* reaches its maximum value for each of the three structures, the volumetric expansion rate of the unit cell structure also reaches its peak values of 388% (SP), 488% (SD), and 394% (GY), respectively. In the calculations, the moment of maximum volume expansion is referenced to the point where 𝜑 = 60° in the bistable state, while the monostable structure is also calculated with 𝜑 = 60° as the reference. Furthermore, we investigated how the initial angle (*θ*) of the rotating unit influences the bistability of the structure as shown in Figure 3d. In both 2D and 3D configurations, the initial angle (*θ*) influence the dimensional constraint factor (𝜹) as shown in Figure S8 (Supporting Information). In the 2D configuration, the range of the rotational angle (𝜑) is 0° to 180°, with 0° and 180° corresponding to the two stable states of the structure. At 90°, δ reaches its maximum value, marking the critical point of the structure. At this critical point, the strain energy of the structure reaches its maximum. Similarly, in the 3D configuration, the rotating unit changes from a quadrilateral to a hexagonal shape, reducing the range of *𝜑* to 0°–60°. The first and second stable states in the 3D configuration occur at 𝜑 = 0° and 𝜑 = 60°, respectively. During the deformation process in the 3D configuration, *θ* not only affects the occurrence of the critical tipping point and the second stable state but also influence the maximum value of *δ*. Specifically, the configuration with an initial angle of 5° achieves a bistable state with less deformation compared to its counterpart with an initial angle of −5°.

Additionally, the dependence of force response to the size of the RVEs used in the numerical simulation is examined. With the RVE expanding from 1 unit cell to 8‐unit cells (2 × 2 × 2), the bistable behavior remains intact for SP kirigami metamaterials, but deteriorates for SD and GY cases (Figure S9, Supporting Information). The mirror symmetry in the kirigami metamaterials helps synchronize the rotations of adjacent hexagon units toward a stable coordinated morphing. Furthermore, the stiffness variation of three types of TPMS‐based solid structures and kirigami designs was systematically compared across different relative densities as shown in Figure S10 (Supporting Information). While kirigami structures generally exhibit lower stiffness than their solid counterparts, they demonstrate superior stiffness tunability enabled by their cut‐guided reconfiguration mechanisms.

Interestingly, a nearly isotropic mechanical response is found for the auxetic GY kirigami metamaterials (shown in Figures S12–S15, Supporting Information). Nearly identical mechanical responses are exhibited when the RVE is stretched along [100], [110] and [111] directions. Zener ratios, which are the characteristics of isotropy for cubic materials, of the GY kirigami metamaterials are calculated with stiffnesses from the initial linear stress‐strain responses. The values are 1.126, 1.054, and 1.068 for different design parameters, all approaching 1, indicating nearly ideal isotropy. Moreover, although only metamaterials with linear mechanical responses have been examined for isotropy in previous reports, our simulations further extend the isotropy concept to metamaterials with nonlinear mechanical behaviors. Since such mechanical isotropy is not observed on SP or SD kirigami metamaterials, we infer that it is inherited from the original gyroid structure which has been reported to exhibit isotropic linear elasticity. The unique symmetry of gyroid is the key to guarantee an isotropic mechanical response even in the nonlinear cases of GY kirigami metamaterials.

### 2.4 Programmable Morphing of Modular Unit‐Cell Assemblies

**Figure 4:**
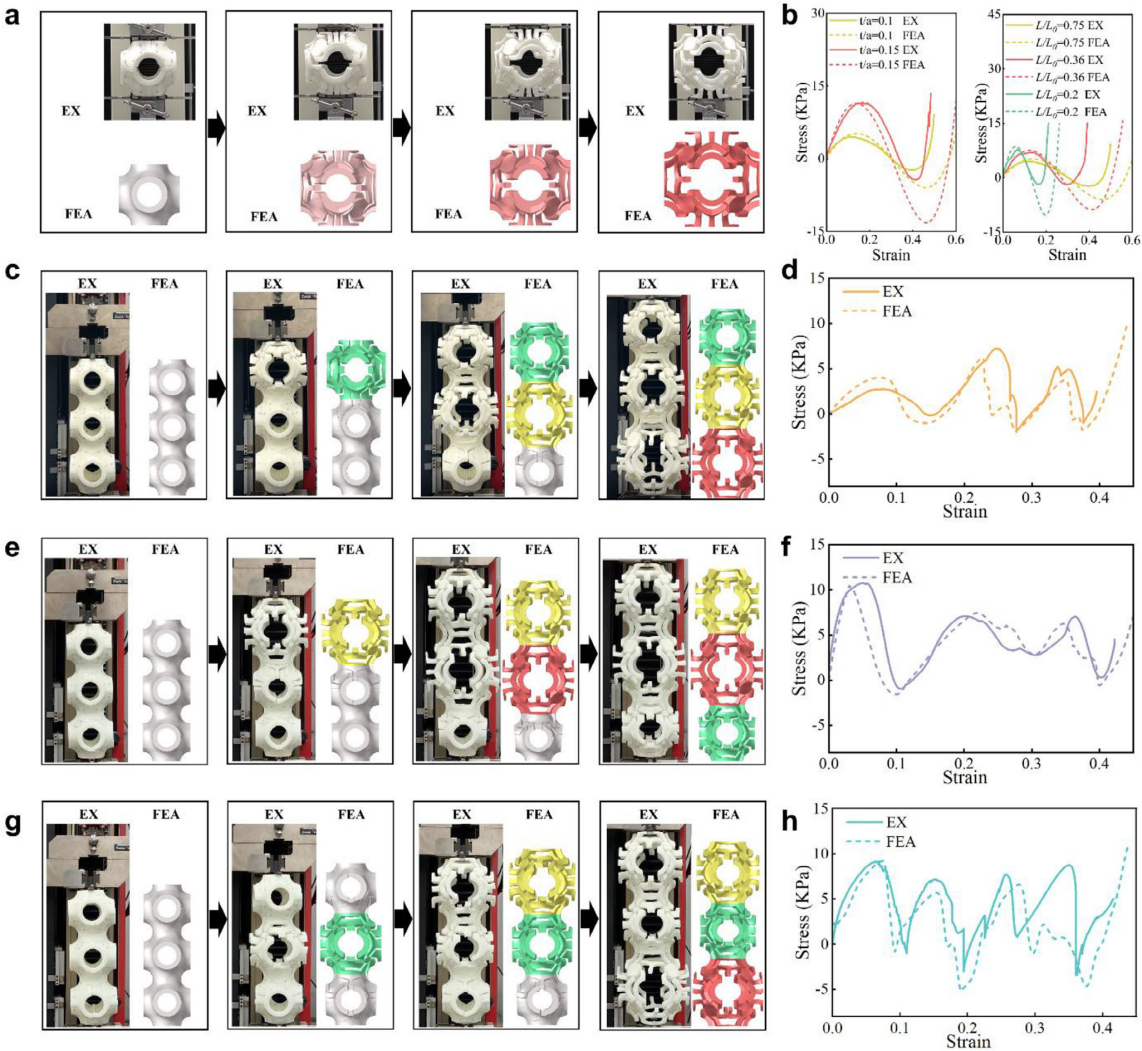
a) The comparison between the experimental and FEA deformed shapes of SP kirigami metamaterials with unit cells under uniaxial tension. Different shades of red represent the state of the structure being stretched. b) Engineering stress‐strain curves of the SP unit cell in experiments (solid line) and finite element simulations (dashed line) are presented with varied *t/a*, *L/L_0_*, and *θ* = 0°. c, e, g) Comparison of the experimental and FEA deformed shapes of three connected SP kirigami unit cells with different *L/L_0_* values for uniaxial tension. The structure in red corresponds to *L/L_0_* = 0.75, yellow to *L/L_0_* = 0.36, and green to *L/L_0_* = 0.2. d, f, h) Engineering stress–strain curves of three connected SP unit cell in experiments (solid line) and finite element simulations (dashed line) are presented with varied *L/L_0_* (t/a = 0.1, θ = 0°). All specimens were pulled at a strain rate of 10 mm min^−1^, and the length of the unit cell edge was 80 mm.

Subsequently, experiments are conducted to verify the FEA results. Differently, parameterized SP kirigami unit cells are additive manufactured and assembled to undergo uniaxial tension tests. **Figure** **4**a shows the tested unit cells and the experiment set‐up for uniaxial tension. It is important to note that the uniaxial tension of such a unit cell is no longer constrained by periodic boundary conditions; as a result, the finite element analysis has been adjusted accordingly. As shown in Figure 4b, the obtained stress‐strain curves represent clearly bistable mechanical responses. The FEA results fit well with the experimental measurement. Volumetric changes during morphing and coordinated rotations of internal elements are observed in detail (Movie S1, Supporting Information). More importantly, the flexibility of our kirigami design allows these unit cells with different force responses to be connected arbitrarily to one another. To realize such a connection, the arm element needs to be printed in an asymmetric way to accommodate different geometries of the RVEs on both sides. Figure 4c,e,g demonstrate three connected SP kirigami unit cells with different *L/L_0_* values. When stretched in a serial manner, the order of deformation differs among the three‐unit cells, as shown in Movie S2 (Supporting Information). During the tensile process, all samples will transition through multiple metastable states before reaching the stable state of full unfolding. The stress‐strain curve displays three prominent peaks, symbolizing the expansion process of the unit cell with varying *L/L_0_* values, while the negative segment signifies the structural bistability, as depicted in Figure 4d,f,h. In a series connection, all unit cells experience the same tensile force, determining the sequence of cell deformation based on their peak stresses prior to deformation. While the peak forces remain relatively consistent during stretching, there is a notable variation in deformation displacement, as illustrated in Figure 4b. That is, a small stretching distance can cause unite cell (*L/L_0_* = 0.20) to deform first, as seen in Figure 4c,g. Figure 4e shows one end of unite cell (*L/L_0_* = 0.20) is fixed, causing the other end with unite cell (*L/L_0_* = 0.36) to expand first. The experimental curve in Figure 4h, in addition to the main peaks of the numerical curve, also shows some larger peaks. This may be due to tiny misalignments and manufacturing defects that occurred during the sample fabrication process, which mildly affected the local snap‐through events. The designed structures consisting of multiple unit cells, exhibit fully reversible deformations between the two bistable states under both tensile and compressive loading. The state transitions are achieved by overcoming an energy barrier, typically manifested as a snap‐through instability. Furthermore, the bistable structures demonstrate excellent mechanical robustness and repeatability, maintaining consistent performance over repeated actuation cycles (as shown in figure S16, Supporting Information). During cyclic loading, the stress‐strain curve forms a hysteresis loop due to energy dissipation caused by internal friction during structural deformation. This energy loss mechanism results in irreversible conversion of a portion of the input mechanical work. In summary, the morphing characteristics of the fabricated cubic metamaterials demonstrate programmable mechanical tunability through controlled voxel arrangements.

**Figure 5:**
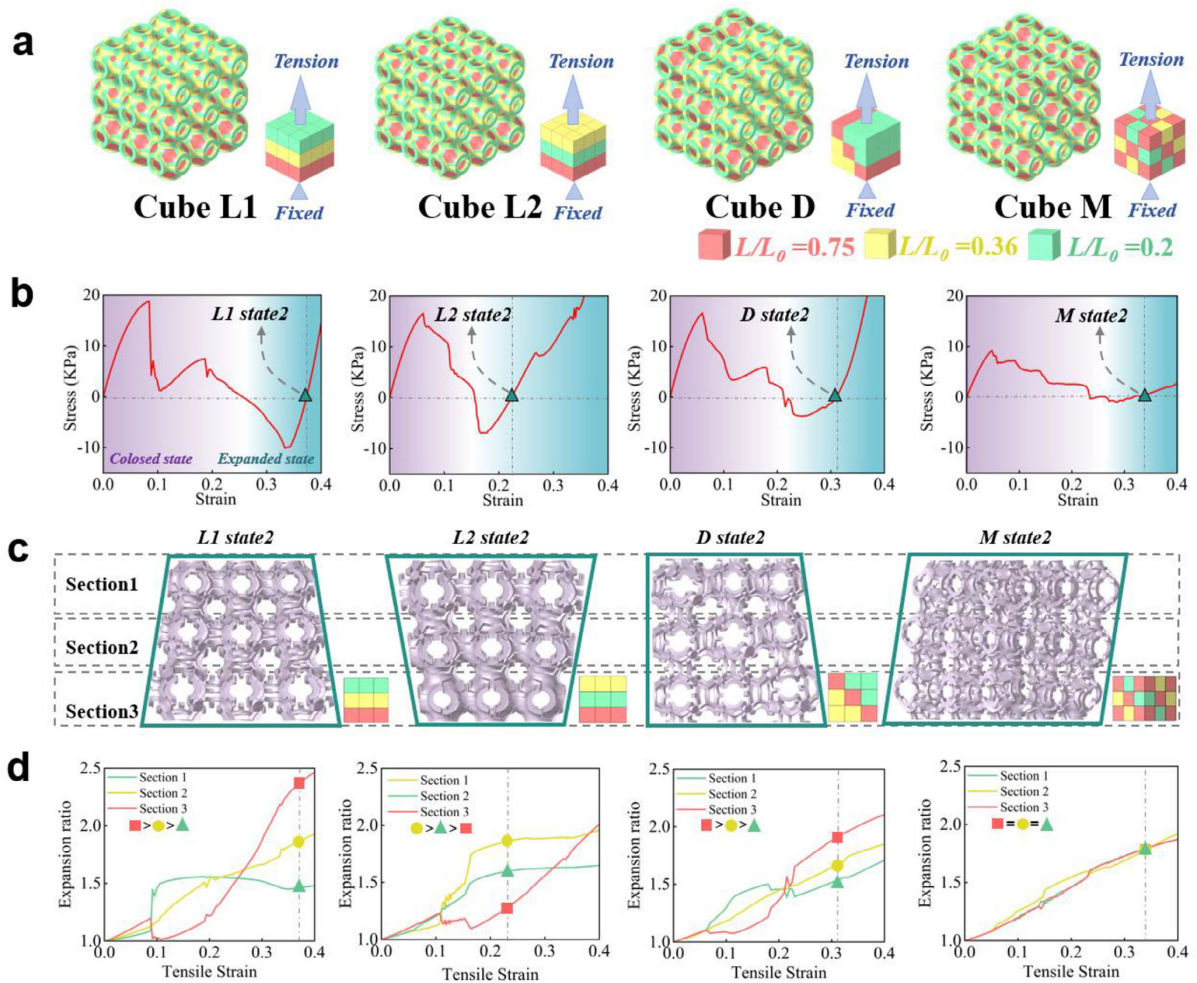
a) Illustrations of the synthesized 3×3×3 voxel cubes (L1, L2, D, M) with three different *L/L_0_* values (0.2, 0.36, and 0.75). b) Stress and strain for the four different combination cubes. During the tensile process, one end is fixed and the other end is stressed. c) The configurations of four synthesized cubes (L1, L2, D, M) when they reach their second stable state during the tensile process. d) The expansion and strain of each layer in L1, L2, D, and M cubes under tension applied to one end.

The programmable morphing order of connected 3D kirigami unit cells inspires artificial metamaterial assemblies of desired multistable mechanical properties. Specifically, each differently parameterized unit cell can be treated as a metamaterial voxel, and many unique voxels can be arranged in spatial order to synthesize new metamaterial bulks. In **Figure** **5**a, we demonstrate several synthesized cubes with 3 × 3 × 3 voxels (SP) of three different *L/L_0_* values (0.2, 0.36, 0.75), similar to the aforementioned uniaxial tensile sample, but more complex in spatial combinations. The synthesized metamaterial cubes exhibited layered (cube L1 & L2), diagonally segregated (cube D) or fully mixed (cube M) voxel arrangements, each cube consists of the same number of voxels from three parameters. Figure 5b depicts the stress‐strain curves of these synthesized metamaterial cubes, including peak/valley stresses and strains. It can be seen that the stress and strain responses of the designed cubes can be flexibly tuned with different voxel arrangements. For example, L1 cube has the highest peak stress, nearly twice as large as compared to the peak stress of M cube, and the lowest valley stress, 11.12 times that of M cube. Meanwhile, the bistable snapping strain of L2 cube is postponed to ≈0.25 compared to other cubes (L1, D, M).

The snapping order of the internal voxels of the cubes can be altered as well. Figure 5c and Figure S17 (Supporting Information) show the configurations of four synthesized cubes (L1, L2, D, M) when they reach their second stable state during the tensile process. Due to the different structural arrangements, these cubes exhibit distinct configurations in the second stable state (Movie S3, Supporting Information). Similar to the L2 structure, the system does not fully deploy when transitioning into the second stable state. This partial deployment behavior stems from the auxetic deformation mechanism of the L2 unit under external loading. Specifically, the constrained expansion of Section 2 (*L/L_0_* = 0.2) mechanically limits the deformation capacity of Section 3 (*L/L_0_* = 0.75), consequently inducing premature bistability in the overall structure. This variation highlights the high degree of programmability in the morphing behavior of 3D kirigami unit cells. Figure 5d shows the horizontal dimension monitoring results of L1, L2, D, and M cubes. The abrupt jumps in the expansion curves mark the occurrences of snapping movements. The major snapping of the L1 cube occurs at a strain of 0.08, while the major snapping of the L2 cube occurs at a strain of 0.15. Due to the greater degree of spatial randomness in the D and M structures, the abrupt changes in horizontal expansion at each layer are less noticeable, particularly in the M cube. It is worth noting that the stress‐strain response of L1 and L2 cubes are rather different from the 1 × 3 uniaxial tensile samples demonstrated in the previous part, although the sequence of the 3 different voxels in these two samples is identical. We infer that the considerable horizontal expansion in the L1 and L2 cubes play an important role in the coordinated morphing of the whole structure. The detailed morphing dynamics in finite or infinite voxelated metamaterial volumes are left for discussion in our future works.

## 3 Conclusion

We have developed a novel class of 3D kirigami metamaterials through the design strategy of hyperbolic tessellations. Three metamaterial designs based on the conjugate SP, SD, and GY structures are respectively introduced. These mechanical metamaterials exhibit unconventional mechanical properties, such as auxeticity, multistability, and programmable morphing capability. The unique harmonious rotations of hinged kirigami elements enable extremal mechanical responses, in particular, the Poisson's ratio can reach down to ‐1. Flexibly tunable stress–strain responses are realized by varying the geometrical parameters of the kirigami metamaterials. The maximum effective volume expansion of the metamaterials after tension can reach up to 388% (SP), 488% (SD), and 394% (GY). Interestingly, a rarely reported isotropic multistability is discovered. Furthermore, a persistent bistable behavior is seen in SP kirigami metamaterials as the number of unit cells in the metamaterial scales, thanks to the synchronized rotation. Synthetic voxelated metamaterial devices with programmable morphing orders and mechanical responses are also demonstrated. The design strategy and metamaterial structures presented in this work advance the frontiers of research on 3D multistable metamaterials. The proposed methodology connecting non‐Euclidean kirigami and real‐world 3D design could inspire rich discoveries of architected materials and impact profoundly the future practicality of metamaterials.

## 4 Experimental Section

### Design and Fabrication

The realization of the design process described above requires the use of computational tools such as Surface Evolver and modeling software like AutoCAD. The three types of TPMSs are obtained through iterative calculations in Surface Evolver, while the thickening and various symmetric operations are completed in AutoCAD (see supporting information of Figure S18, Supporting Information). The specimens were fabricated using SZUV‐W8006 photosensitive resin via photopolymerization‐based additive manufacturing (3DSL‐800, SHDM, China) The printing layer height and speed are 0.1 mm and 7500 mm/s, respectively. The unit cell edge was printed with a length of 80 mm. The entire structure is printed in three parts (one rotating section and two connected arms) and then assembled together through the hinge section.

### Finite Element Analysis

The finite element simulations were conducted using the commercial software ABAQUS (SIMULIA), with the Explicit solver applied to model the rapid dynamic deformation of the bistable structure. The metamaterial was discretized using C3D8R hexahedral elements (average element size: 1.68 mm) with assigned material properties.

A mesh convergence study confirmed the numerical reliability, as demonstrated in Figure S18 (Supporting Information). A mesh convergence study confirmed the numerical reliability, as demonstrated in Figure S19 (Supporting Information) The base material selected with an elastic modulus E = 1.3 GPa and a Poisson's ratio ν = 0.3. The loads and boundary conditions in the finite element simulations were configured to match the experimental conditions, ensuring consistency and accuracy in the results.

### Mechanical Measurements

Uniaxial loading tests were conducted on the SP structure to characterize the mechanical properties of the metamaterial. Displacement‐controlled loading was applied using a universal testing system (Zwick/Roell Z1.0, Zwick, Germany). The specimens were connected to sliding blocks at both the top and bottom ends of SP, with the bottom firmly secured to a sliding rail as shown in Figure S20. The top end was also connected to a sliding rail, allowing the loading head to be pulled upward at a rate of 10 mm min^−1^.

## Conflict of Interest

The authors declare no conflict of interest.

## Supporting information



Supporting Information

Supporting Information

Supplemental Movie 1

Supplemental Movie 2

Supplemental Movie 3

## Data Availability

The data that support the findings of this study are available from the corresponding author upon reasonable request.
