# Supplementary material for: 3D Hyperbolic Kirigami Metamaterials With Tunable Auxeticity and Multistability
Source: Adv Sci (Weinh). 2025 Jun 23;12(35):e06703. doi: 10.1002/advs.202506703 (PMC12463087; doi:10.1002/advs.202506703)
Supplement: Supplementary file 1 — Supporting Information [file ADVS-12-e06703-s004.docx]

Supporting Information

3D Hyperbolic Kirigami Metamaterials with Tunable Auxeticity and Multistability

Yu Lei, Yan Wang, Ruizhi Cui, Xiaolong Huang, Lei Zhang, Yuan Jin*, Jinling Gao*, Biwei Deng*

In hyperbolic geometry, regular polygonal tilings of type {p,q} consist of congruent regular p-gons such that q polygons meet at each vertex ^[1,2]^. When the inequality (p−2) (q−2)>4 holds, such a tiling can only exist in hyperbolic space. The tiling {6,4}, where four regular hexagons meet at every vertex, is one such example. To represent the hyperbolic plane, we adopt the hyperboloid model:

(S1)

With the Lorentzian inner product:

(S2)

The group of isometries preserving this inner product, SO^+^(2,1), acts transitively on and is used to construct regular tiling.

For visualization, we map the hyperboloid model to the Poincaré disk model via：

(S3)

With the inverse:

(S4)

To construct the {6,4} tiling, we begin by defining a fundamental domain in , typically a regular hyperbolic hexagon. Using a discrete subgroup $\Gamma\in\mathrm{SO}^{+}(2,1)$, the space is tiled as:

(S5)

where F is the initial tile. This tiling is visualized in via the projection. Mapping relationship between Hyperboloid model and Poincaré Disc model as shown in Figure S1 (a).

Additionally, we introduced the geodesic to split the hyperbolic polygons. These are hyperbolic geodesics-curves of minimal distance-that connect specific pairs of points on the polygon boundary ^[3]^. The hyperbolic bistable kirigami metamaterials are inspired from the bistable kirigami in , the kirigami is implemented based on geodesics that connect polygon edges at different equal points, the rotation angle and scaling mentioned in the article are also achieved by changing the positions of different equal points.

The initial angle of a model with a predetermined scaling ratio can be controlled through parameters α and β, as defined in the equation.

(S6)

Conversely, the initial scaling ratio of a model with a fixed rotation angle can be adjusted using parameters γ and η.

(S7)

As illustrated in Figure S1(b), curve Euclidean segments in correspond under projection to hyperbolic geodesics in TPMS in .

*
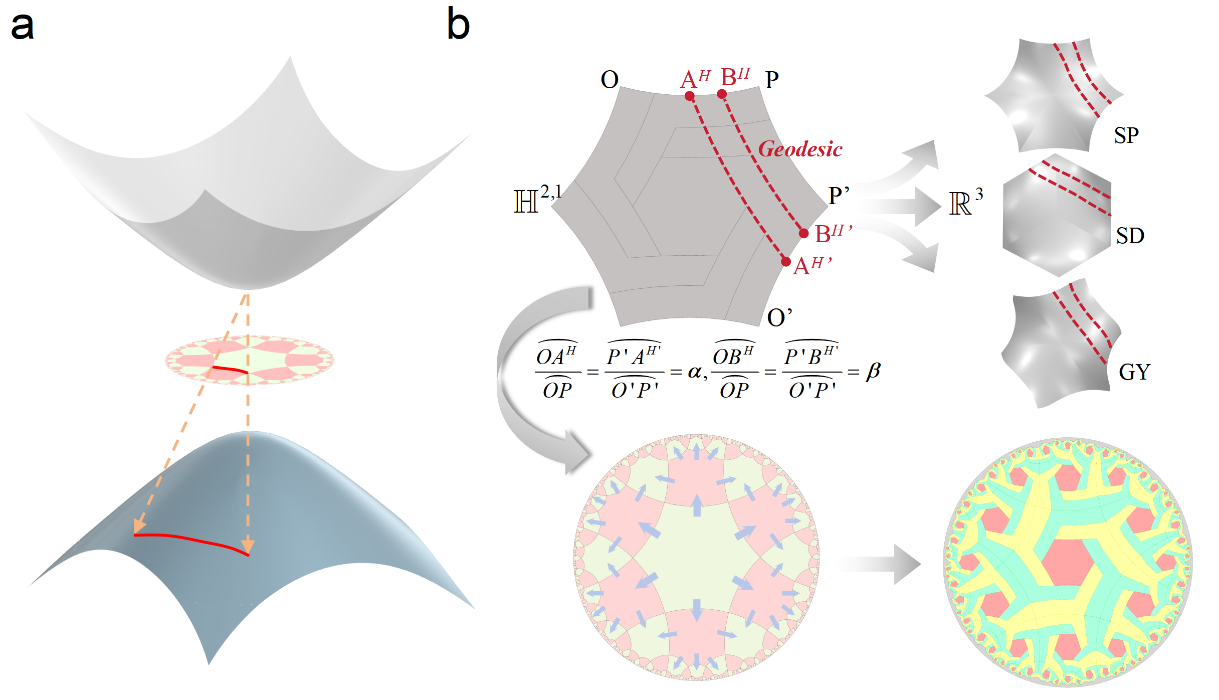
*

**Figure S1** (a) Mapping relationship between Hyperboloid model and Poincaré Disc model. (b) Methodology of kirigami for Euclidean polygon and Hyperbolic polygon in Poincaré Disc.

In 2D configurations, the process of bistability is the process of breaking the line L_ABCD_ from the restriction of the line L_AB_, and the second stable state is the moment when the line _LABC’D’_ is symmetric to the line L_ABCD_ about L_AB_ as shown in Figure S2 (a). Under quasi-static tension, when L_ABC’D’_ is in the green region, external work is stored in the structure in the form of strain energy in the system. If the strain in the structure is small or the material's yield strain is high, it will be fully elastic strain energy. At this stage, removing the load will release the stored strain energy and restore it to the first stable state. Elastic strain energy will be completely released in the pink region, and when reaching the second stable state, the elastic strain energy will be completely released, and the internal energy of the system will be zero as shown in Figure S2 (b). Continuing to apply the load will continue to charge the system, and removing the external force before the material buckles will cause the structure to return to the second stable state.


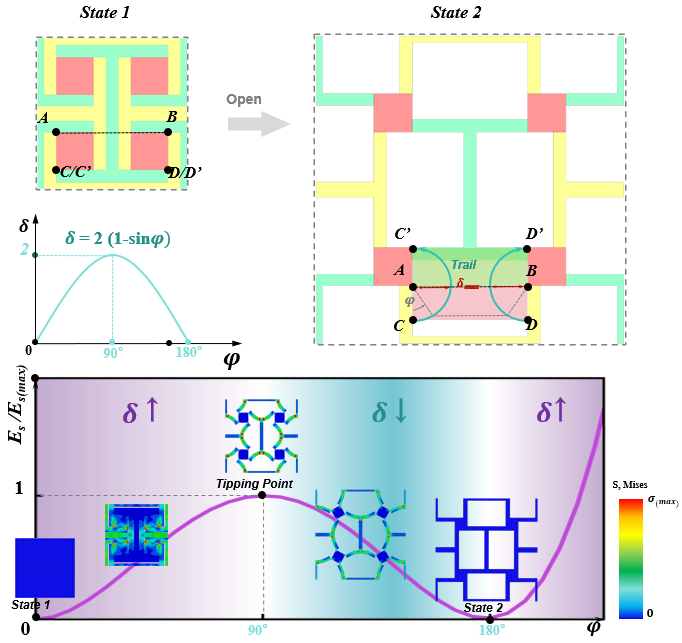


**Figure S2** (a) Schematic diagram of the 2D rotational rigid configuration expanding from stable state 1 to stable state 2, (b) Schematic diagram of the relationship between the ratio of strain energy and the rotation angle φ.

**Table 1.** The sizes of these angles in the three configurations (SP, SD, and GY)

| **Angle/deg°** | $\boldsymbol{\alpha}_{\boldsymbol{1}}$ | $\boldsymbol{\beta}_{\boldsymbol{1}}$ | $\boldsymbol{\alpha}_{\boldsymbol{2}}$ | $\boldsymbol{\beta}_{\boldsymbol{2}}$ |
| --- | --- | --- | --- | --- |
| SP | 54.18 | 30 | 54.18 | 30 |
| SD | 20.98 | 30 | 17.28 | 32.68 |
| GY | 5.33 | 33.91 | 36.58 | 47.17 |

The multistable characteristics exhibited by the SP, SD, and GY depend on the spatial evolution configuration. Under equal-biaxial stretching, individual units undergo clockwise or counterclockwise rotation, resulting in the expansion of the original structure. For example, the configuration of the SP is primarily achieved through the mirror symmetry of the unit structure (eight cells) as shown in Figure S3 (a). The SD configuration is mainly formed through the 180° rotation of the unit structure (four cells) along the three-dimensional axis as shown in Figure S3 (b). The orientation of each rotating unit is consistent, resulting in a reduction of symmetry information, consequently, a weakening of bistable characteristics. While, the Gyroid has no planes of symmetry and no embedded straight line. The central rotating unit of the eight cells rotates consistently, the adjacent rotating units connected to it rotate in the opposite direction, as shown in Figure S3 (c). During stretching, the orientation of each rotating unit opposes that of the adjacent rotating unit, as shown in Figure S3 (d).


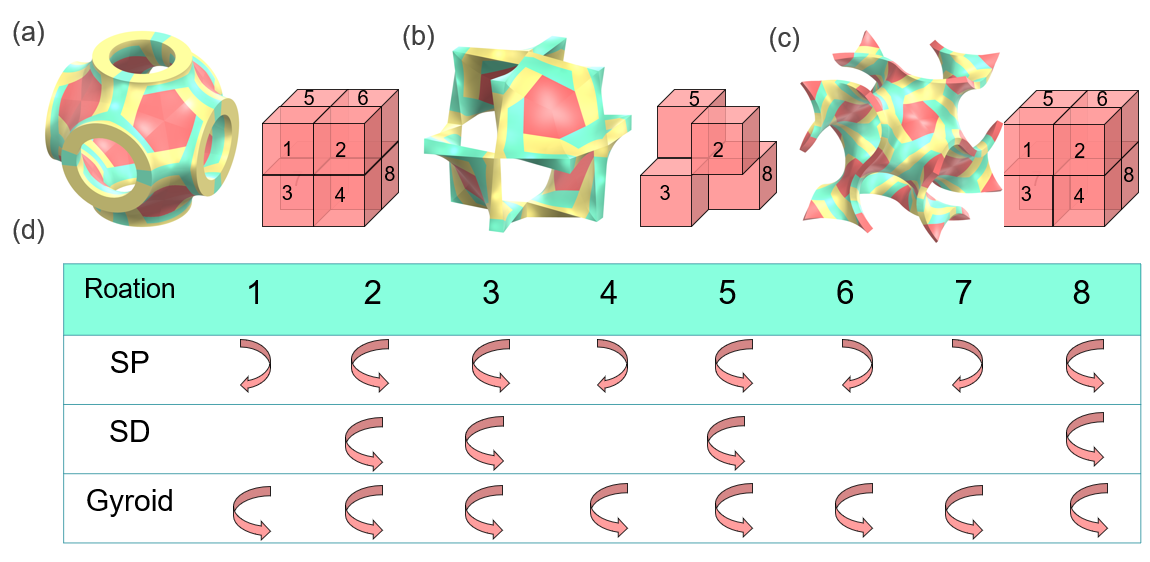


**Figure S3**. (a), (b) and (c) The unit cell of the SP, SD, and Gyroid are assembled from one-eighth of the basic unit; A cube is divided into 8 smaller cubes. (d) Rotation directions of complete rotating rigid bodies with the SP, SD, and Gyroid during the stretching process.

The stress distributions in the SP, SD, and GY structures at the maximum dimensional constraint factor (δ = δ_max_), corresponding to the state of peak stress. Notably, the stress distribution in the hinge regions is highly non-uniform, with maximum stresses reaching 37.2 MPa (SP), 80.4 MPa (SD), and 103.9 MPa (GY), as shown in Figure S4 (a-c). The simulations were conducted using the SZUV-W8006 photosensitive resin, which has a Young’s modulus (E) of 1600 MPa and a tensile strength of approximately 62 MPa. As observed, the maximum stresses in the SD and GY structures exceed the material’s tensile strength. It is important to clarify that this study primarily focuses on structural design and mechanical mechanisms. To isolate the influence of material properties on the structural performance, the simulations were performed under idealized linear elastic conditions, without incorporating plastic properties or collapse models. Therefore, while the stress distributions in SD and GY reflect the structural response under these idealized assumptions, they do not represent the actual plastic deformation or failure behavior that would occur in physically printed models under real-world conditions.


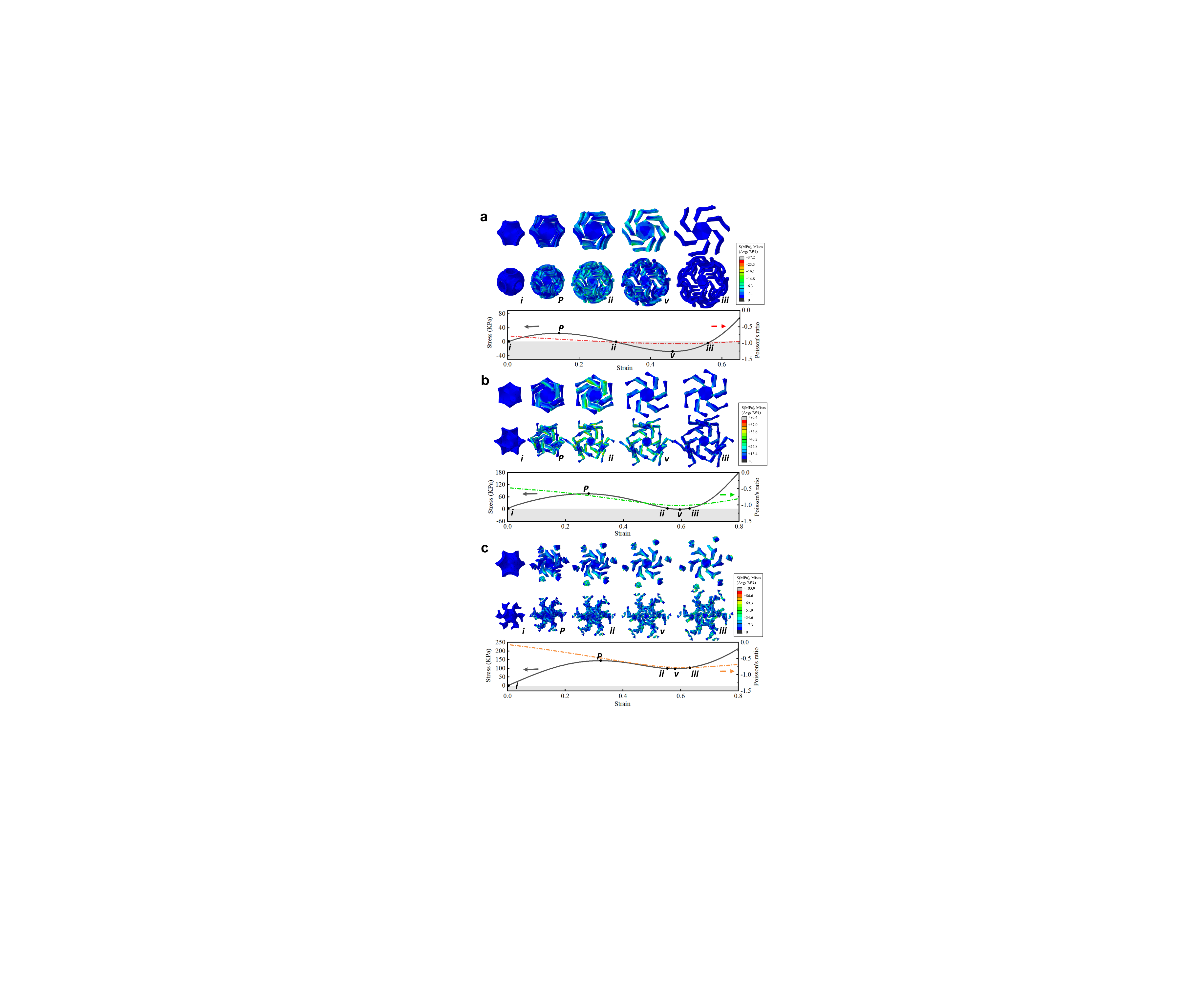


**Figure S4**. Mechanical response of multistable auxetics to uniaxial loading. (a-c), FE (solid lines) stress-strain curves during loading (tension) and characterized Poisson’s ratio (dashed lines) for SP (red) (t/a = 0.1, L/L_0_ = 0.75, θ = 0°), SD (green) (t/a = 0.1, L/L_0_ = 0.50, θ = 0°) and GY (yellow) (t/a = 0.1, L/L_0_ = 0.24, θ = 0°), respectively. The insets in the figure represent the auxetic states at different stages (ⅰ, P, ⅱ, v, and ⅲ). The upper illustrations are enlarged views of a local region from the lower illustrations, showing the stress distribution of the minimal unit (comprising six connecting arms and a hexagonal rotating part) during the auxetic deformation process under varying strain conditions.

The stress distributions within the SP, SD, and GY structures under the maximum dimensional constraint factor (δ = δ_max_), corresponding to the peak stress state. To replicate realistic structural conditions, a hinge-based connection model was constructed, as illustrated in Figure S5a, where each hinge contains a stainless-steel pin insert (Figure S5b). Finite element analysis was performed under experimentally relevant loading conditions to identify the critical stress region, which was determined to be the interface between the rotating component and the pin. The von Mises stress at this critical interface was extracted, as shown in Figure S5c. The maximum stress occurs when the dimensional constraint factor δ reaches its peak (corresponding to a tensile strain of 0.28), with a value of 37.04 MPa (Figure 5d). This value is well below the tensile strength of the resin material used in the experiments (50 MPa), thereby satisfying the structural strength requirements. The simulations were conducted using SZUV-W8006, a commercial photosensitive resin with a Young’s modulus of 1600 MPa and a tensile strength in the range of 50–60 MPa.


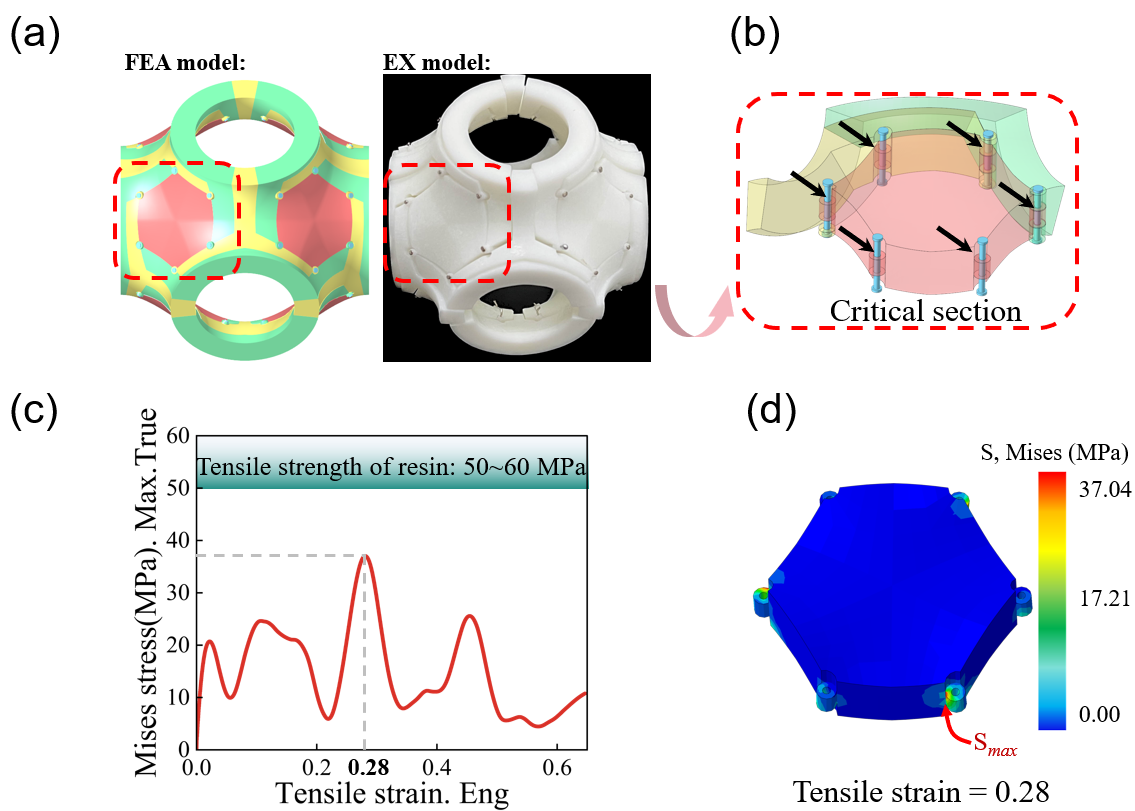


**Figure S5**. (a) Schematic of the SP structural model for simulation and additive manufacturing, featuring rotating units interconnected through hinge joints. (b) Magnified view of the hinge region marked in (a). (c) Stress and tensile strain at the critical interface during auxetic deformation of the structure. (d) Stress distribution on the rotating assembly, with localized stress concentration observed at the pin-connection regions.


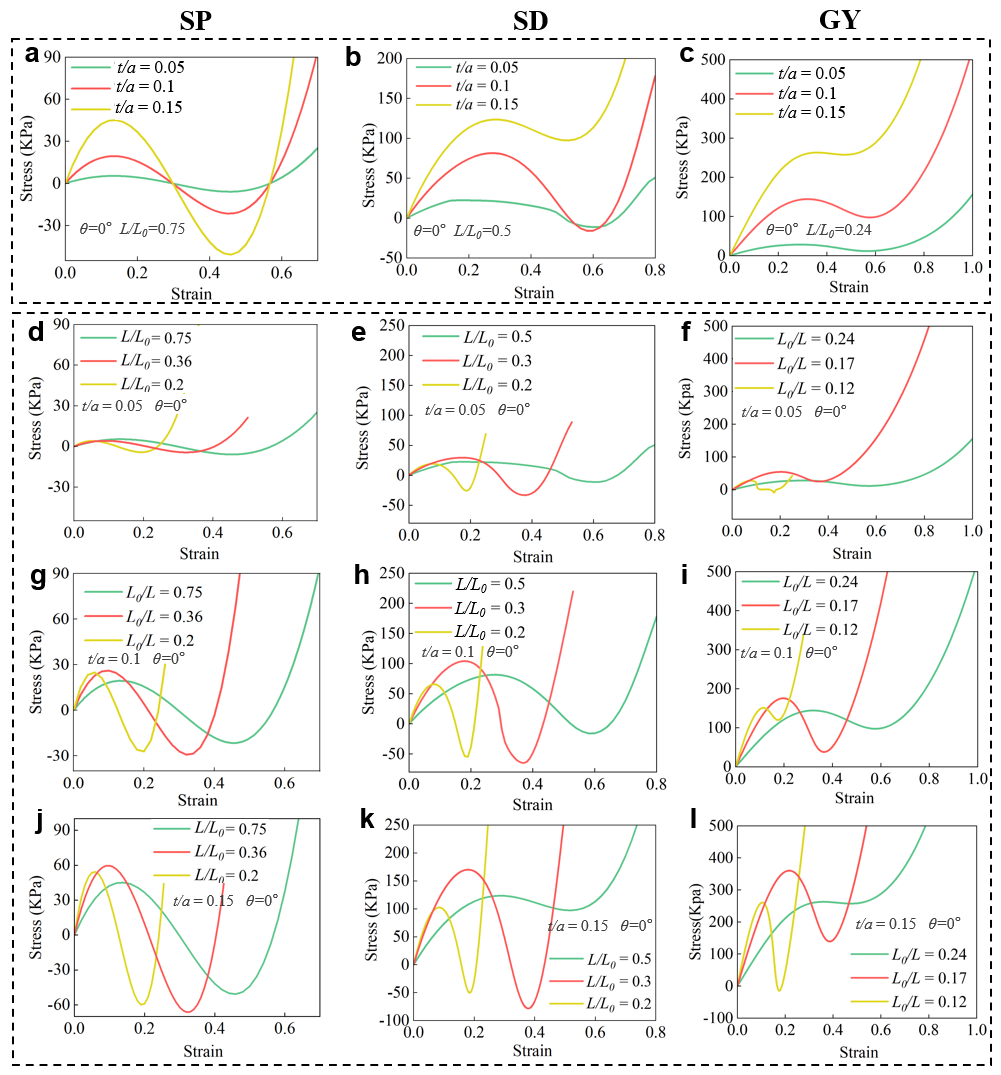


**Figure S6.** The uniaxial stress-strain curves for the SP, SD, and GY kirigami metamaterials, with different values of *t/a* (a-c) and *L/L_0_* (d-l).

An increase in the t/a ratio results in the amplification of both local maximum and minimum stresses during bistable morphing. This phenomenon occurs because thicker structures store greater elastic energy in their rotating arms during deformation. During hinge rotation of a finite-thickness arm, the rotational degree of freedom about the x-axis (Rx) generates a bending moment (Mx), while rotation about the y-axis (Ry) produces a torsional moment (My) as shown in Figure S7. These two moments are primarily responsible for the non-uniform axial stress distribution observed in the hinge regions. The remaining degrees of freedom contribute differently: translations along x and y directions induce uniform normal stresses, translation along z causes uniform shear stress, and rotation about z (Rz) does not generate any stress.


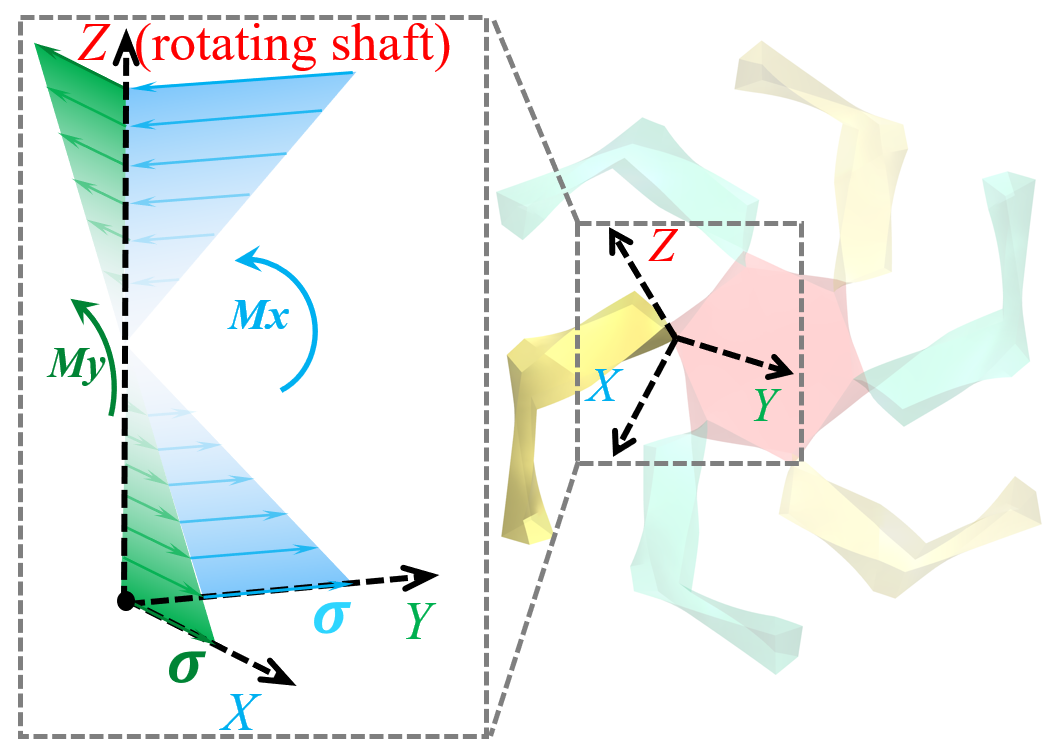


**Figure S7.** The stress differences caused by wall thickness during the rotational process of the hexagonal rigid-body structure.


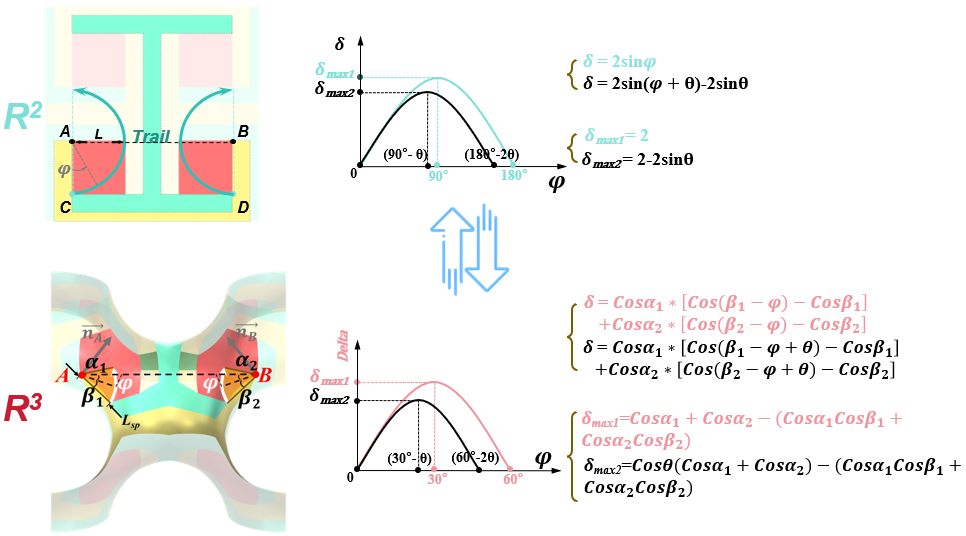


**Figure S8** The influence of the initial angle *θ* on the dimensional constraint factor is observed in both 2D and 3D configurations.


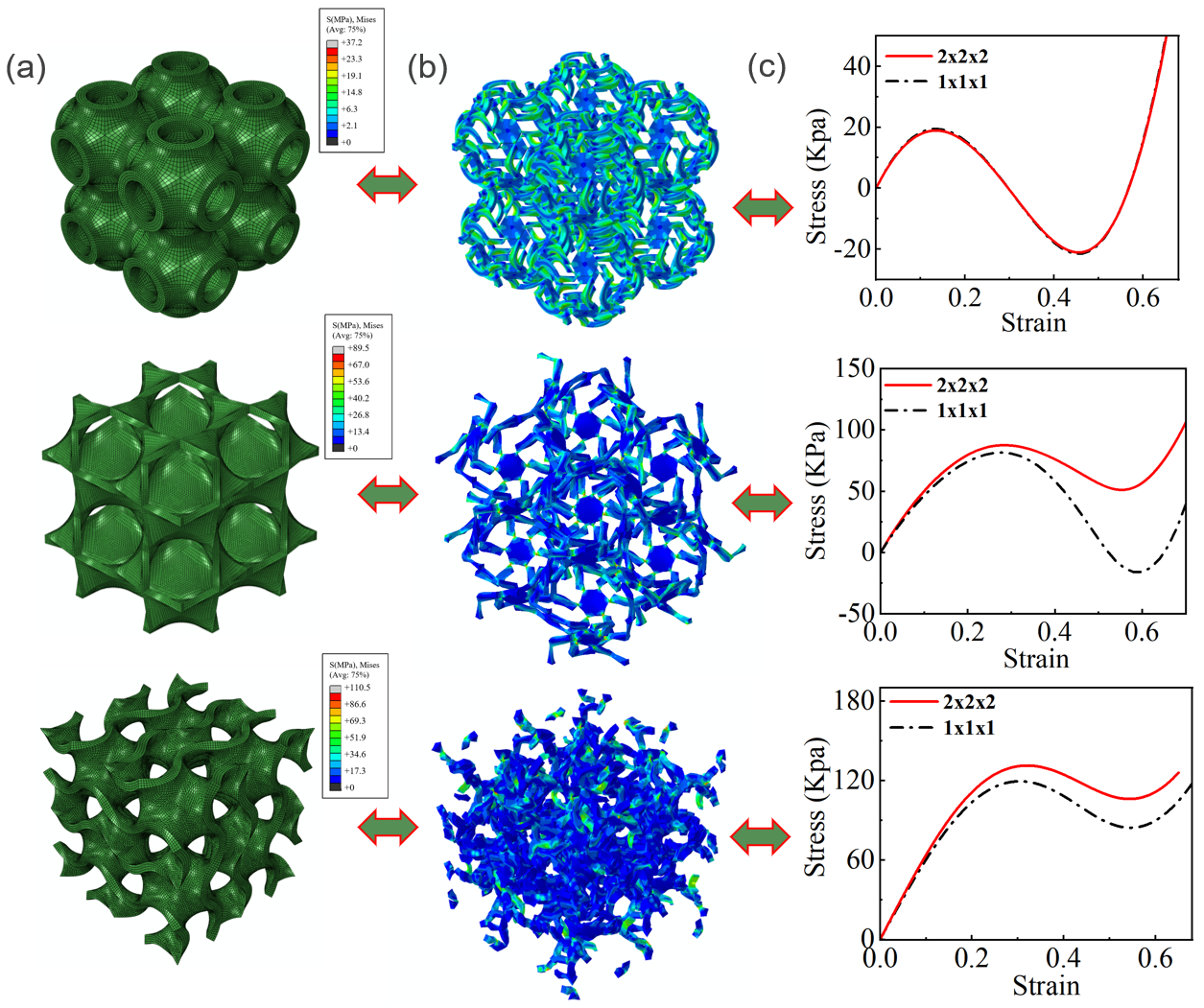


**Figure S9**. (a) Multicellular of the SP (t/a=0.1, θ=0°, *L/L_0_*=0.75), SD (t/a=0.1, θ=0°, *L/L_0_*=0.50), and GY (t/a=0.1, θ=0°, *L/L_0_*=0.24) with a 2x2x2 cells. (b) Multicellular of the SP, SD, and GY under uniaxial stretch until complete expansion. (c) Stress and strain for the SP, SD, and Gyroid by FEA result under periodic boundary conditions.


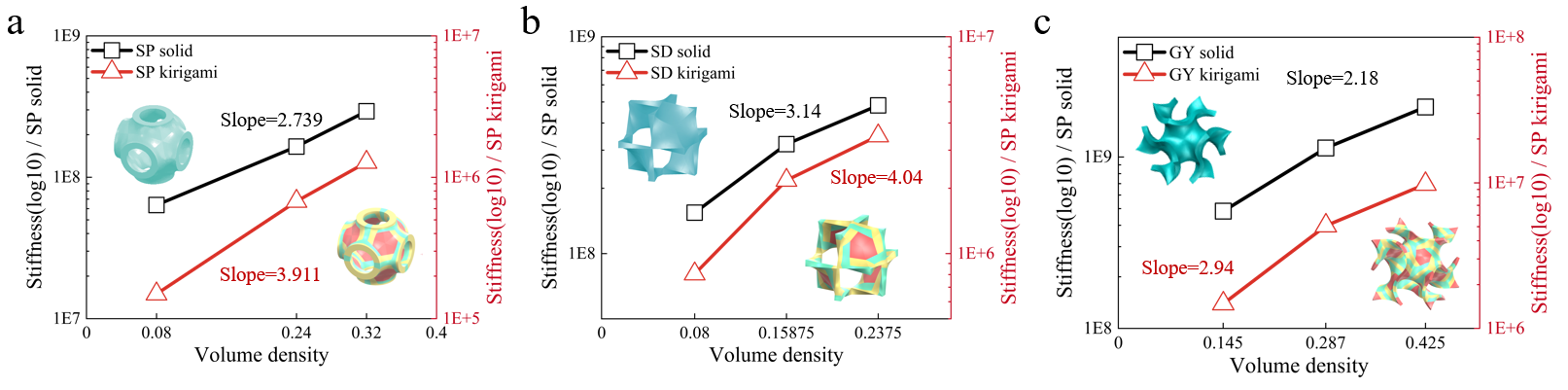


**Figure S10** The stiffness values of (a) SP, (b) SD, and (c) GY structures in both solid and kirigami configurations at varying relative densities.

To explore the unique characteristics of the gyroid structure, this study applied loads along three lattice orientation (100), (110), and (111). Finite element analysis was conducted based on normal and shear strains to obtain the corresponding stress-strain curves, as illustrated in Figure S11. The stress-strain curves for the gyroid in three different directions with varying design parameters were found to exhibit remarkable proximity. This suggests that, even when subjected to changes in thickness and scaling ratio conditions, the gyroid structure maintains isotropy, showing approximate isotropy with similar elastic moduli in different directions.


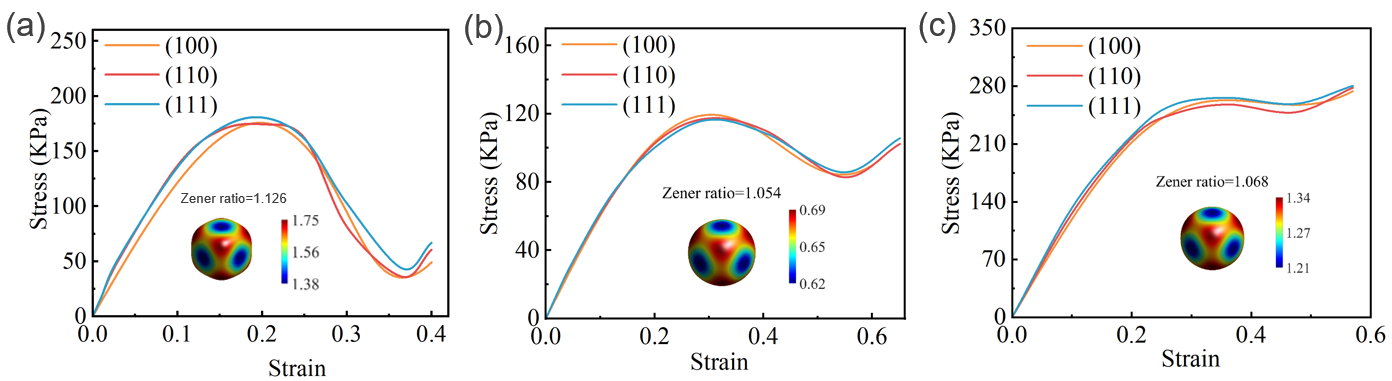


**Figure S11**. Stress-strain curves for the three directions of the Gyroid structure with parameters *t/a* =0.1, *L/L_0_* =0.17, *θ* = 0° (a); *t/a* =0.1, *L/L_0_*=0.24, θ = 0° (b); *t/a* =0.15, *L/L_0_*=0.24, *θ* = 0° (c).

To examine the directional mechanical responses of SP, SD, and GY kirigami metamaterials, we performed comparative simulations of these structures under loading along three lattice orientations: (100), (110), and (111). Sizes of the RVEs subject to periodic boundary conditions in the simulations are also varied to further probe the validity of the simulation results. Models of a single unit cell, 2x2x2 unit cells and 3x3x3 unit cells are built. As illustrated in Figure S12 (a-b), both SP and SD structures exhibit anisotropic behaviors in both linear and nonlinear regimes. Such anisotropic behaviors are observed under model configurations of both single unit cell and 2x2x2 unit cells. Notably, the SP models exhibit identical mechanical responses between the two configurations. This is because the morphing of SP kirigami is highly coordinated among every rotating unit, thanks to the unique mirror symmetry. This leads to a stable anisotropic response from SP kirigami metamaterials. In contrast, when the RVE of the model extend from a single cell to multiple unit cells, SD and GY kirigami exhibited changed stress-strain relationships. This is due to the non-uniform morphing across the entire RVE while mirror symmetry is no longer enforced. Interestingly, the nearly mechanical isotropy of GY kirigami is barely affected by the size of the RVE. As shown in Figure S12 c), very close nonlinear mechanical responses of the GY kirigami is observed when loaded along (100), (110), and (111) directions for RVEs of a single unit cell, 2x2x2 unit cells and 3x3x3 unit cells. This validated our hypothesis that the mechanical isotropy of GY kirigami is originated from symmetry-based rules, despite the nonlinear mechanical response and non-uniform morphing behavior


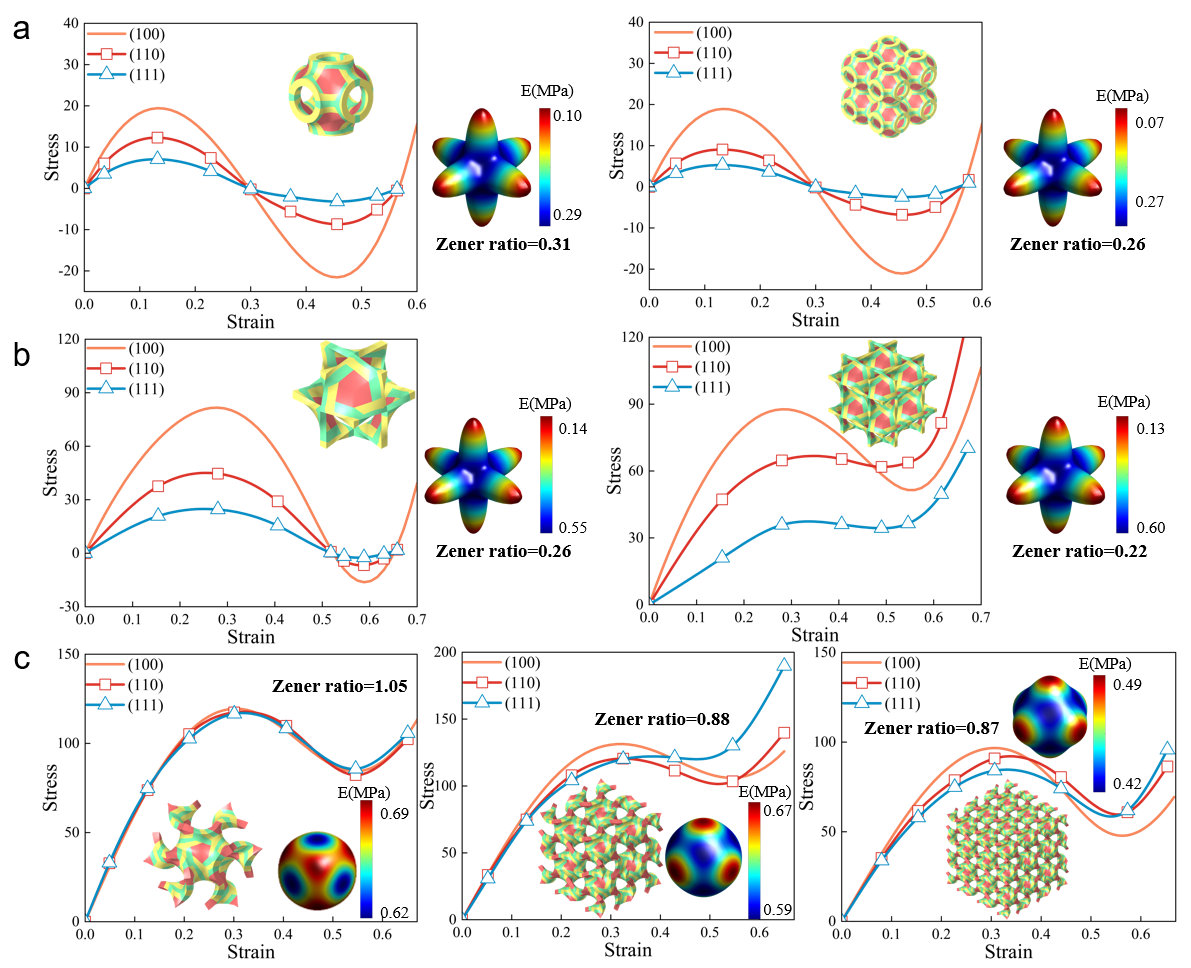


**Figure S12**. Stress–strain curves in three directions for the SP (a), SD (b), and GY (c) structures with the following parameters: (a) t/a = 0.1, L/L_0_ = 0.75, θ = 0°; (b) t/a = 0.1, L/L_0_ = 0.50, θ = 0°; (c) t/a = 0.1, L/L_0_ = 0.24, θ = 0°. The inset figures depict the value of Zener ratio.

Figure S13 (a) shows the uniaxial tensile strain in the (100) direction as the displacement boundary condition. Because the structure's Poisson's ratio changes with the increase of tensile strain during the stretching process, this kind of uniaxial tensile state can be considered equivalent to the strain state shown in Figure S13 (b). Where $\varepsilon_{x}=\varepsilon,\varepsilon_{y}=\varepsilon_{z}=\nu\varepsilon$, In which, $\nu=\nu(\varepsilon)$. $\varepsilon_{x}$, $\varepsilon_{\text{y}}$and $\varepsilon_{\text{z}}$ represent the principal strain in the three axial directions, $\nu$ represents Poisson's ratio, $\varepsilon$ represents the strain values corresponding to points A, B, C, D, E, F, and G in Figure S13 (c), respectively. Seven representative points on the stress-strain curve in the (100) direction of the gyroid structure show in Figure S13 (c). These correspond to seven strain values achieved during uniaxial tension, which can be respectively equated to strain states with simultaneous application of normal strain and shear strain, that is, the strain states in the (110) and (111) directions.


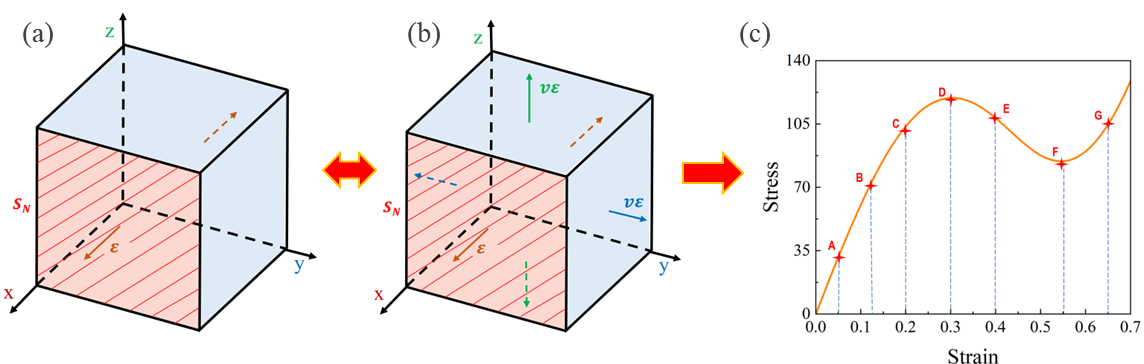


**Figure S13**: (a) and (b) Schematic equivalent stress interchange diagram on the surface of an element, (c) The stress-strain curve of the gyroid structure in the (100) direction.

When calculating the stress-strain in the (110) direction, the z-axis direction is not considered, and the strain value is still $\nu\varepsilon$as shown in Figure S14 (a). Therefore, when performing an equivalent transformation at a 45° direction, the strain state is as shown in Figure S14 (b). According to the deformation compatibility equation:

$\varepsilon_{\text{（}1,1,0\text{）}}=\varepsilon$ (S8)

$\varepsilon_{\text{（-}1,1,0\text{）}}=\nu\varepsilon$ (9)

Which can be obtained $\varepsilon_{x}=\varepsilon_{y}=1/2(1+\nu)\varepsilon$ (S10)

$\left|\gamma_{\text{xy}}\right|=\left|\gamma_{\text{yx}}\right|=1/2(1-\nu)\varepsilon$ (S11)

$\gamma_{\text{xy}}$and$\gamma_{\text{yx}}$represent the shear strain in the x and y axis directions. Under the constraint of displacement boundary conditions, the reactionary forces in the equivalent strain states for these seven points are calculated. Their force states are shown in Figure S14 (b). Therefore, according to the equilibrium equation of forces: $\sum F_{x}=\sum F_{\text{y}}=0$, the force in the (110) direction can be determined as:$f_{N}=\sqrt{2}/2(f_{x}+f_{xy}+f_{y}+f_{yx})$, and thus the equivalent stress is:$\sigma_{N}=f_{N}/S_{N}(S_{N}=\sqrt{2}L^{2})$.$f_{x}$, $f_{\text{y}}$and $f_{xy}$, $f_{yx}$ represent the principal force and shear force in the x and y axis directions, respectively. $f_{N}$ represents the principal force in the (110) direction, $S_{N}$ represents the area of ABCD in the (110) direction. The edge length of the cube is defined as L.

When calculating the isotropy in the (111) direction, the strain state is as shown in the Figure S15. According to the deformation compatibility equation:

$\varepsilon_{\text{（}1,1,1\text{）}}=\varepsilon$ (S12)

$\varepsilon_{\text{（}1,-1,0\text{）}}=\nu\varepsilon$ (S13)

$\varepsilon_{\text{（}1,1,-2\text{）}}=\nu\varepsilon$ (S14)

The strain state of principal strain and shear strain can be determined:

$\varepsilon_{X}=\varepsilon_{Y}=\varepsilon_{Z}=1/3(1+2\nu)\varepsilon$ (S15)

$\gamma_{XY}=\gamma_{YZ}=\gamma_{XZ}=1/3(1-\nu)\varepsilon$ (S16)

Based on the displacement boundary conditions, the reaction forces under the equivalent strain states for these seven points are calculated, and their loading conditions are shown in Figure S15. The direction cosines in the (111) direction can be expressed as:

$l=\cos(N,x)=\sqrt{3}/3$ (S17)

$\text{m}=\cos(N,y)=\sqrt{3}/3$ (S18)

$\text{n}=\cos(N,z)=\sqrt{3}/3$ (S19)

Therefore, according to the equilibrium equation of forces: $\sum F_{x}=\sum F_{\text{y}}=\sum F_{\text{z}}=0$

Which can be obtained

$\text{p}_{x}=lf_{x}+mf_{yx}+nf_{zx}$ (S20)

$\text{p}_{\text{y}}=mf_{y}+nf_{zy}+lf_{xy}$ (S21)

$\text{p}_{\text{z}}=nf_{z}+lf_{xz}+mf_{yz}$ (S22)

Substituting into the formula,$f_{N}=lp_{x}+mp_{y}+np_{z}$ we can obtain:

$f_{N}=l^{2}f_{x}+m^{2}f_{y}+n^{2}f_{z}+2mnf_{yz}+2nlf_{yz}+2lmf_{xy}$ (S23)

The equivalent stress in the (1,1,1) direction is:

$\sigma_{N}=f_{N}/S_{N}$ (S24)

$S_{N}=3\sqrt{3}L^{2}/4$ (S25)

$S_{N}$ represents the area of ABCDEF in the (111) direction.


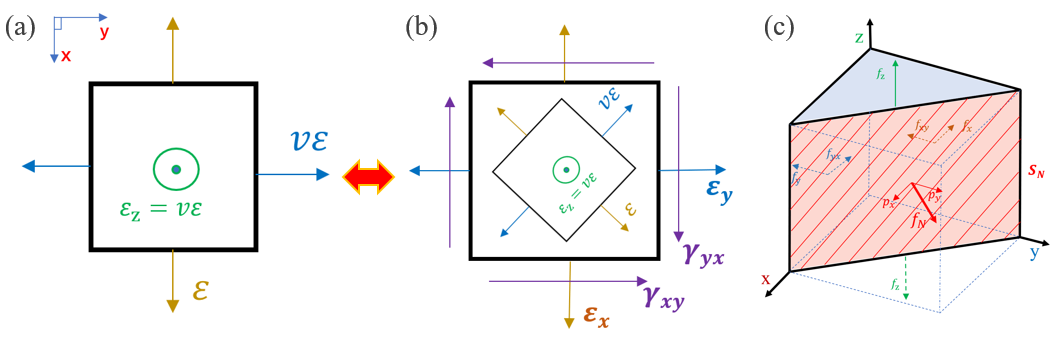


**Figure S14**: (a) and (b) Schematic equivalent stress interchange diagram on the surface of an element, (c) the stress state of the surface (110).


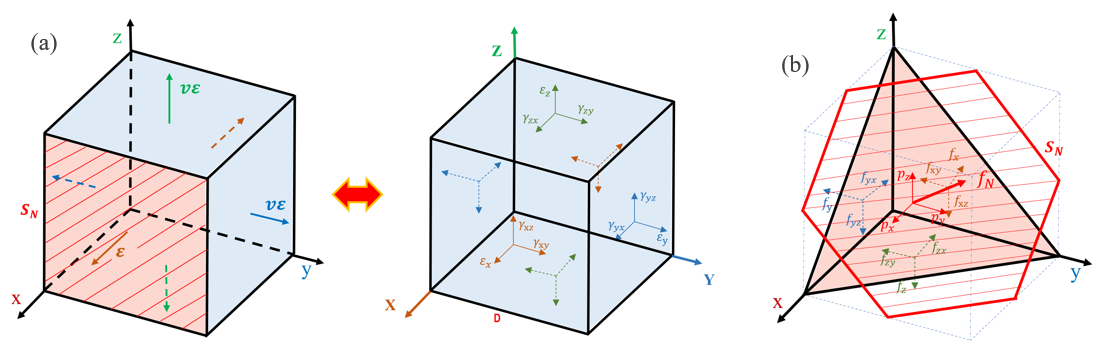


**Figure S15**: (a) Schematic equivalent stress interchange diagram on the surface of an element, (b) the stress state of the surface (111).


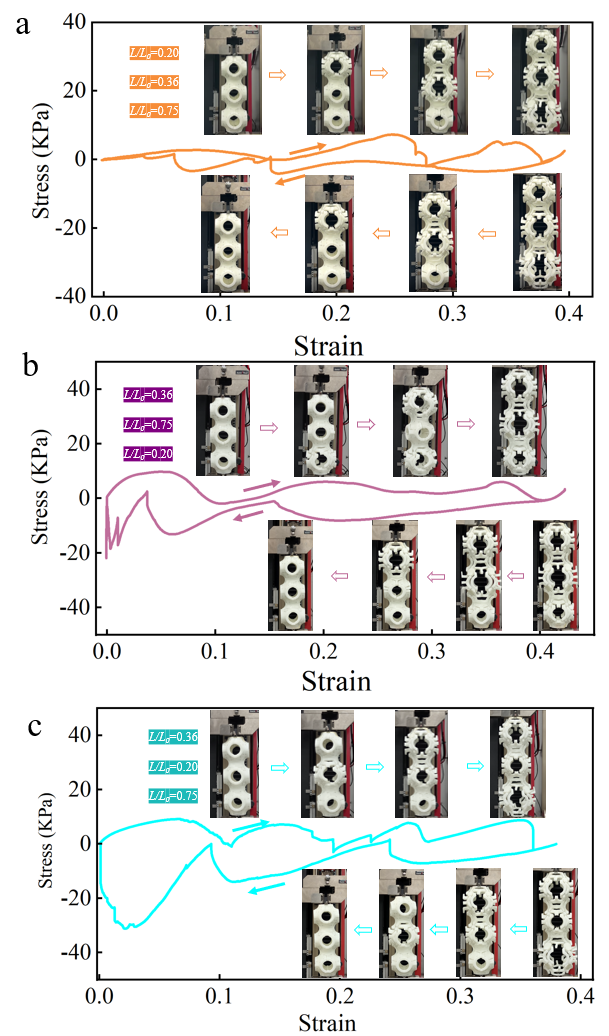


**Figure S16** (a-c) Engineering stress-strain curves of three connected SP unit cell in compressive and auxetic experiments are presented with varied L/L_0_ (t/a=0.1, θ=0°). Corresponding insets illustrate the SP mulit cell configurations at different auxetic and closed stages. All specimens were pulled at a strain rate of 10 mm min−1, and the length of the unit cell edge was 80mm.

Figure S17 systematically examines the deformation behavior of 3×3×3 voxel-based (SP) metamaterial cubes under external loading, focusing on three distinct configurations: layered (L1, L2), diagonally segregated (D), and fully mixed (M). The spatial arrangement of voxels results in markedly different hierarchical expansion characteristics during auxetic deformation. To clarify the dynamic progression toward the second stable state, Figure S17 a) presents structural transformations at five key deformation stages (①–⑤). As shown in Figures S17 b) and c), the layered configurations (L1 and L2) display a sequential, layer-by-layer expansion. Notably, the deformation of each layer is mechanically influenced by adjacent layers. In particular, the middle layer of the L2 cube, composed of voxels with the smallest rotational rigid-body dimensions, shows limited expansion. This constraint hinders the full deployment of the upper and lower layers, leading to premature bistability in the overall structure. By contrast, the D and M configurations exhibit synchronized deformation across layers due to their more homogeneous voxel distributions as shown in Figures S17 d) and e). The M configuration, in particular, undergoes uniform expansion without abrupt transitions in strain. These distinct deformation pathways result in four unique macroscopic morphologies at the second stable state, clearly demonstrating the programmable morphing capabilities of 3D kirigami-inspired metamaterials.


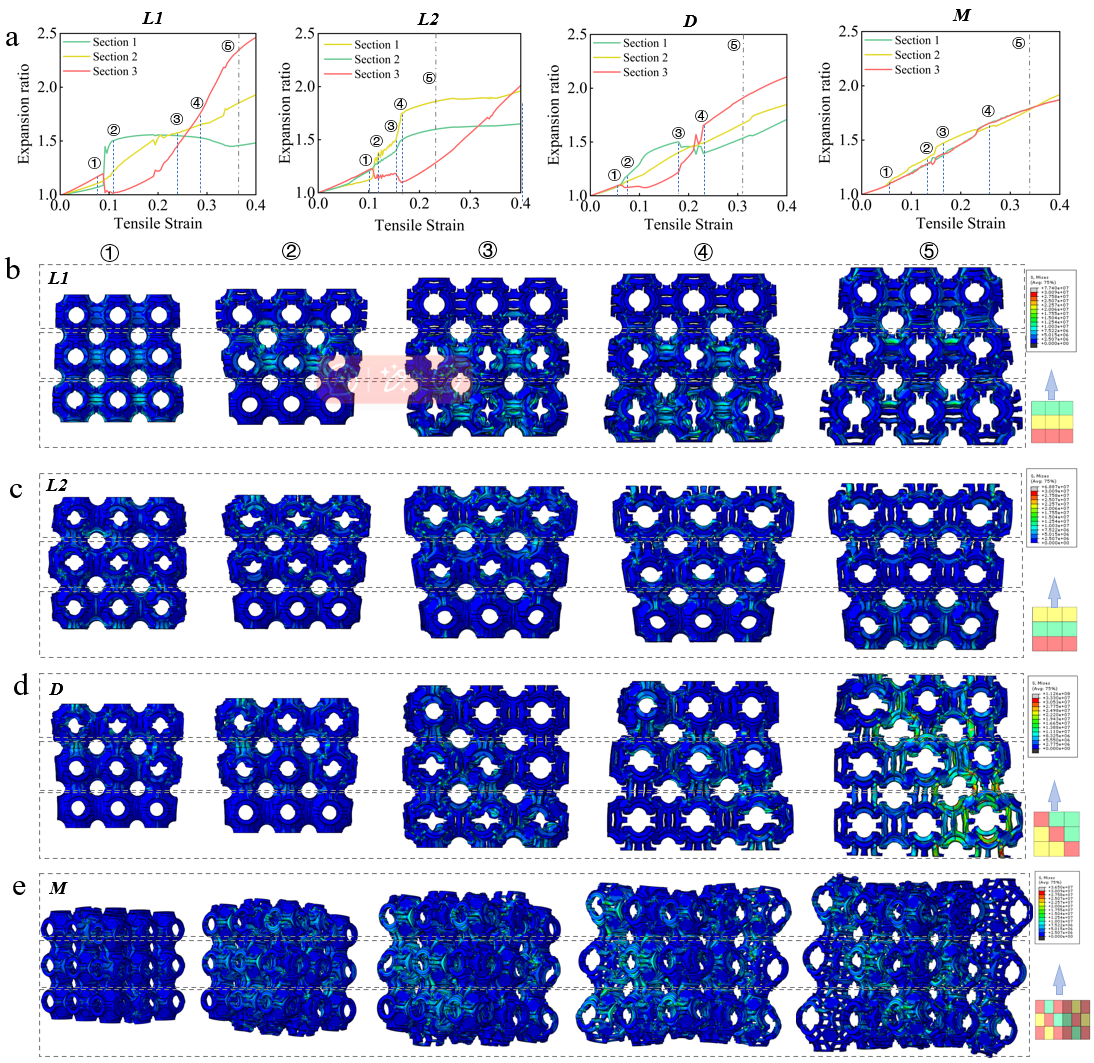


**Figure S17**. a) Expansion and strain distribution in each layer of the L1, L2, D, and M cubes under uniaxial tension applied to one end. b-e) Deformation behavior of the L1, L2, D, and M cubes under external loading, corresponding to the five key deformation stages (①–⑤) depicted in (a).

The process begins by setting up the boundary conditions necessary for generating minimal surfaces in Surface Evolver as shown in Figure S18. This leads to the formation of a grid representation of the minimal surfaces through iterative meshing. Subsequently, these grid-based minimal surfaces are imported into AutoCAD, where the processes of thickening, applying symmetry operations, and segmentation are carried out, resulting in a three-dimensional multistable configuration based on minimal surfaces.


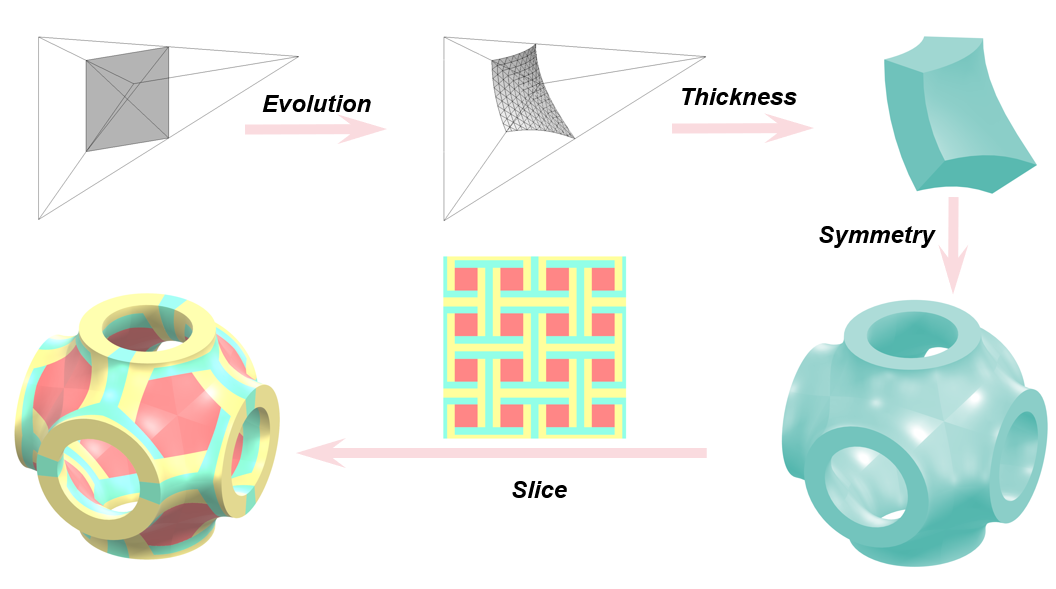


**Figure S18** The operational steps for the design of three dimensional multistable mechanical metamaterials based on minimal surfaces.

A systematic mesh convergence study was performed to verify the numerical independence of our simulation results. The analysis was conducted through progressively refined meshes, spanning an appropriately wide range of element densities from coarse to fine discretization. The finite element mesh discretization of the model is presented in Figure S19 a). The convergence of key mechanical metrics-including engineering modulus and stress-strain response—was quantitatively evaluated, as illustrated in Figure S19 b) and c). The results demonstrate that the engineering modulus stabilizes (variation < 0.29% between 24960 and 79296 elements) beyond a threshold mesh density, confirming solution convergence. Balancing computational efficiency with numerical accuracy, the final simulations adopted an optimized mesh density of 24960 elements (average size: 1.68 mm)


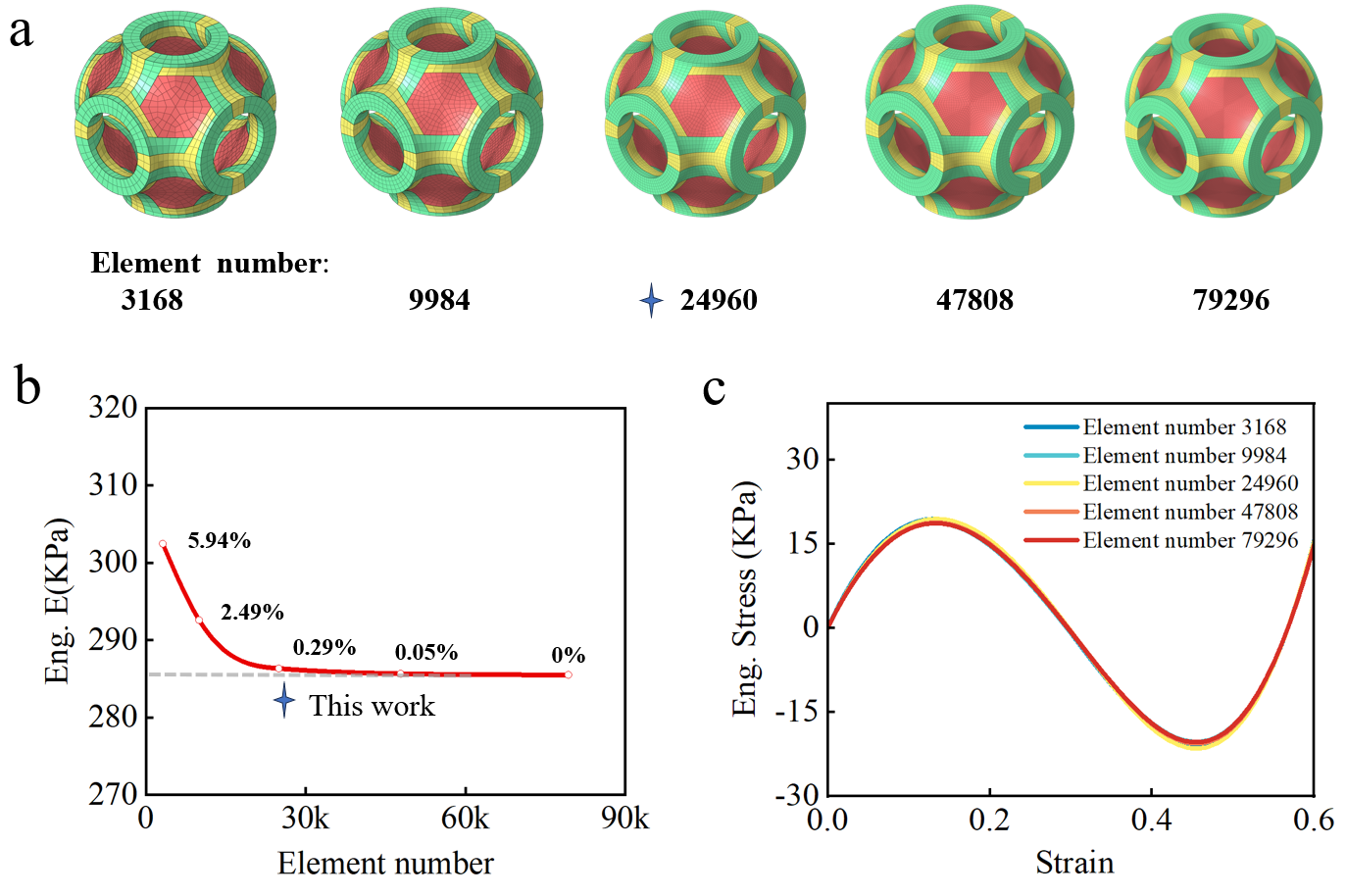


**Figure S19** a) The finite element mesh discretization of the SP model (t/a=0.1, L/L0=0.75, and θ=0°); b) and c) The dependence of both engineering modulus and stress-strain behavior on mesh density.


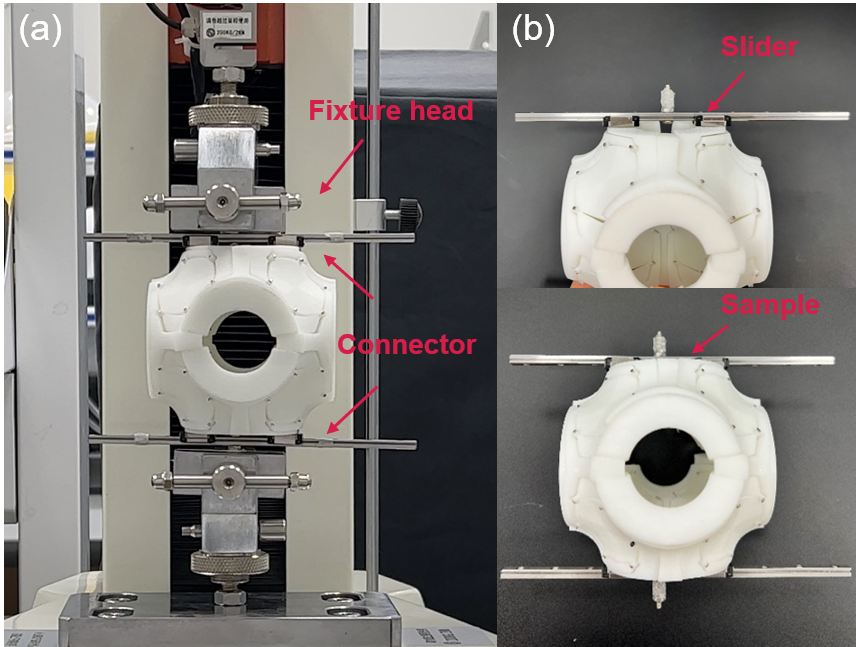


**Figure S20** The experimental setup of the uni-axial loading tests. (a) The uniaxial loading test. (b) The sample, top and bottom connector.

Supplemental Movies

Supplemental Movie S1. Experimental setup and finite element simulation results of unit cells under uniaxial loading.

Supplemental Movie S2. Experimental setup and results of three-unit cells arranged in series under uniaxial loading.

Supplemental Movie S3. Volumetric expansion of multi-cell cubic structures under uniaxial tension.

References

[1] T. Coulbois, D. Pellicer, M. Raggi, C. Ramírez, F. Valdez, *Adv. Geom*. **2015**, *15*, 77.

[2] S. Yao, D. Pi, J. Chen, *Neurocomputing* **2022**, *480*, 119.

[3] L. Bao, Y. Wang, X. Song, T. Sun, *Knowl. Inf. Syst.* **2024**, *67*, 661.
